# Diversity and distribution of polyphagan water beetles (Coleoptera) in the Lake St Lucia system, South Africa

**DOI:** 10.3897/zookeys.656.11622

**Published:** 2017-02-14

**Authors:** Matthew S. Bird, David T. Bilton, Renzo Perissinotto

**Affiliations:** 1 DST/NRF Research Chair in Shallow Water Ecosystems, Nelson Mandela Metropolitan University, C/o Department of Zoology, P.O. Box 77000, Port Elizabeth, 6031, South Africa; 2 Current address: Department of Zoology and Entomology, University of Fort Hare, Alice, 5701, South Africa; 3 Marine Biology and Ecology Research Centre, Plymouth University, Drake Circus, Plymouth PL4 8AA, United Kingdom

**Keywords:** Afrotropical region, biodiversity census, aquatic Coleoptera, Polyphaga, Hydrophilidae, iSimangaliso Wetland Park

## Abstract

Water beetles belonging to the suborder Polyphaga vary greatly in larval and adult ecologies, and fulfil important functional roles in shallow-water ecosystems by processing plant material, scavenging and through predation. This study investigates the species richness and composition of aquatic polyphagan assemblages in and around the St Lucia estuarine lake (South Africa), within the iSimangaliso Wetland Park, a UNESCO World Heritage Site. A total of 32 sites were sampled over three consecutive collection trips between 2013 and 2015. The sites encompassed a broad range of aquatic habitats, being representative of the variety of freshwater and estuarine environments present on the St Lucia coastal plain. Thirty-seven polyphagan taxa were recorded during the dedicated surveys of this study, in addition to seven species-level records from historical collections. Most beetles recorded are relatively widespread Afrotropical species and only three are endemic to South Africa. Samples were dominated by members of the Hydrophilidae (27 taxa), one of which was new to science (*Hydrobiomorpha perissinottoi* Bilton, 2016). Despite the fauna being dominated by relatively widespread taxa, five represent new records for South Africa, highlighting the poor state of knowledge on water beetle distribution patterns in the region. Wetlands within the dense woodland characterising the False Bay region of St Lucia supported a distinct assemblage of polyphagan beetles, whilst sites occurring on the Eastern and Western Shores of Lake St Lucia were very similar in their beetle composition. In line with the Afrotropical region as a whole, the aquatic Polyphaga of St Lucia appear to be less diverse than the Hydradephaga, for which 68 species were recorded during the same period. However, the results of the present study, in conjunction with those for Hydradephaga, show that the iSimangaliso Wetland Park contains a high beetle diversity. The ongoing and future ecological protection of not only the estuarine lake itself, but also surrounding freshwater wetlands, is imperative and should be taken into consideration during future management planning for the park.

## Introduction

A recent survey of the Hydradephaga of the Lake St Lucia system, located within the iSimangaliso Wetland Park, KwaZulu-Natal, South Africa, has shown that this is a hot-spot of aquatic beetle diversity, with 68 species recorded in total, including several new records for the region (Perissinotto et al. 2016). This paper details the polyphagan water beetles found during the course of the same surveys.

The suborder Polyphaga includes the vast majority of beetles, with an estimated 320 000 species currently described in 151 families (Beutel and Leschen 2005). On a global scale, the diversity of Polyphaga that inhabit truly aquatic environments is slightly above that of the Hydradephaga, although in the Afrotropical region more Adephaga have been described to date (Jäch and Balke 2008). Worldwide, thirteen families of Polyphaga have predominantly aquatic representatives, with the Hydrophilidae, Hydraenidae, Scirtidae and Elmidae comprising the bulk of them (Jäch and Balke 2008). Other predominant aquatic families occurring in southern Africa within the suborder are Helophoridae (largely confined to the Palearctic Region, with only two species of *Helophorus* Fabricius, 1775, known from Africa), Epimetopidae (represented in Africa by only one genus, *Eupotemus* Ji & Jach, 1998), Heteroceridae (23 known southern African species), Hydrochidae (with eleven species of *Hydrochus* Leach, 1817, represented in southern Africa), Ptilodactylidae (in a state of taxonomic disarray, but with at least two genera in southern Africa), Spercheidae (monogeneric family with five species recognized in southern Africa), Dryopidae (represented in southern Africa by three genera and eight species); and Psephenidae (with three monospecific genera currently recognized in southern Africa) (Stals and de Moor 2007). Other polyphagans that have affinity for aquatic habitats but are not regarded as true water beetles are now referred to as “paraquatic” (sensu Jäch and Balke 2008). These include the “shore beetles”, “facultative water beetles” and “parasitic water beetles” (sensu Jäch 1998).

Like the hydradephagans, polyphagans are also found in all types of aquatic habitats and although they do not spread into the open ocean, some species are able to tolerate hypersaline conditions as high as 250‰, especially hydraenids in the genus *Ochthebius* Leach, 1815 (Perkins 1980, Gerdes et al. 1985, Abellán et al. 2007). Most species of aquatic Polyphaga are either scavengers or phytophages, but some of the larger species are predatory, particularly in the Hydrophiloidea, which also frequently have aquatic larvae, some of which may be semi-terrestrial (Beutel and Leschen 2005). Thus they play key roles in aquatic ecosystems and may significantly impact the trophic structure and functioning of wetland ecosystems, such as Lake St Lucia and its associated wetlands in northern KwaZulu-Natal, South Africa.

The St Lucia lake system is part of the iSimangaliso Wetland Park, South Africa’s first UNESCO World Heritage Site and a RAMSAR Wetland of International Importance (Perissinotto et al. 2013). The broad region of the park has historically experienced drastic shifts in climatic conditions, with droughts and floods alternating at semi-decadal cycles (Perissinotto et al. 2013). The last wet phase was recorded in the park from 2011 to 2014, resulting in repeated flood events, with large amounts of freshwater flowing into the system, changing the prevailing salinity state from hypersaline to oligo- or polyhaline. This led to the penetration of numerous brackish and freshwater species into the estuarine lake from adjacent rivers and wetlands. Prominent among these were aquatic insects, especially beetles, thereby providing an opportunity and necessity to renew efforts towards the investigation of the diversity and dynamics of this invertebrate component within the system. Here we report the findings of a census on the aquatic Polyphaga undertaken within Lake St Lucia and its associated wetlands during the period July 2013–February 2015. The results of this have been combined with historical records to provide a baseline for future identification and monitoring of beetle biodiversity patterns in response to climatic and anthropogenic changes.

## Materials and methods

The sampling design and protocol for this study follow those described by Perissinotto et al. (2016), who provide a detailed description of study sites and sampling techniques. A summary of their account is provided here.

### Study area

**Figure 1.**
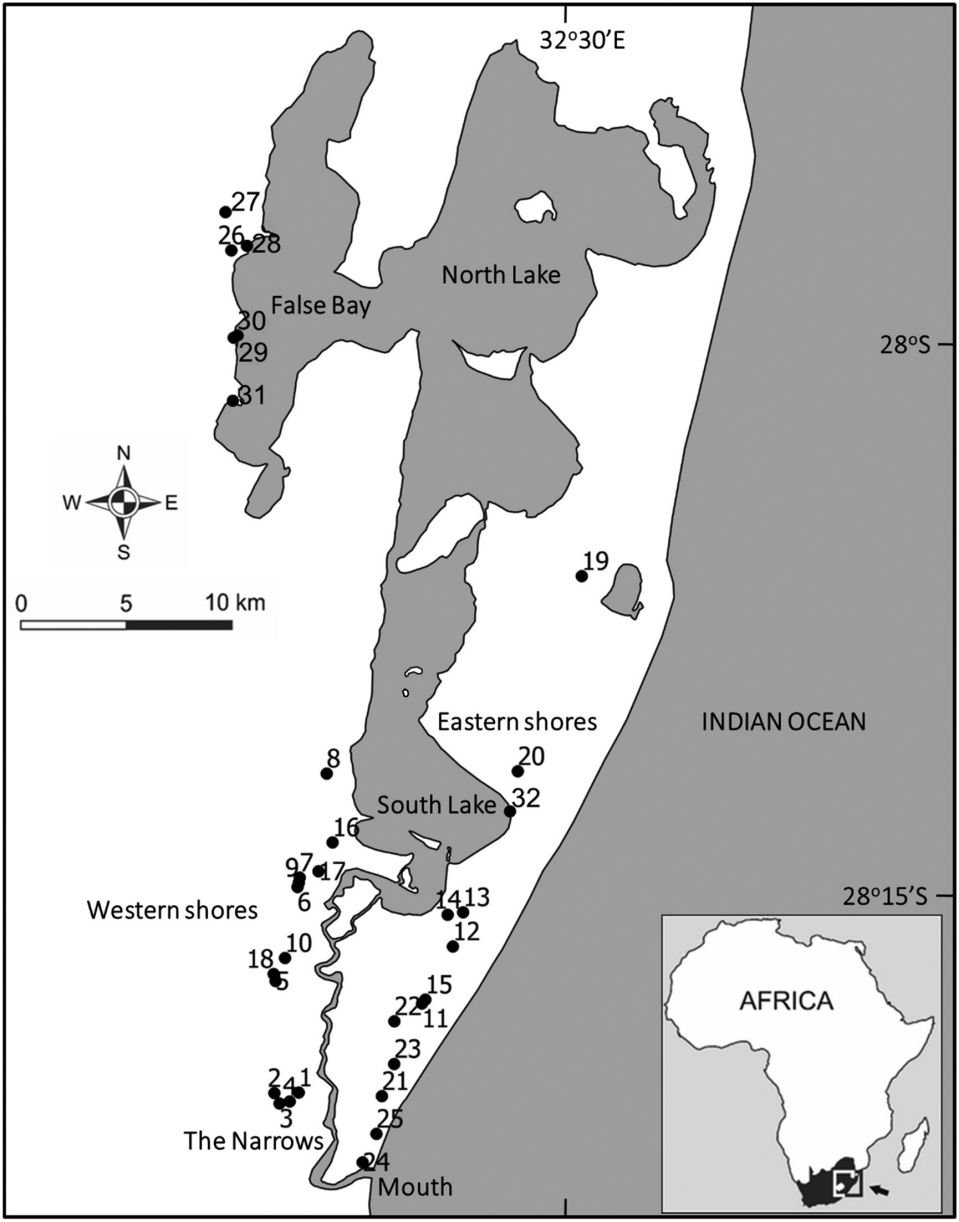
The Lake St Lucia system in northern KwaZulu-Natal. The locations of sites sampled between November 2013 and February 2015 are depicted. Site numbers 1–32 correspond to those in Table 1. Figure reproduced with permission from Perissinotto et al. (2016).

Lake St Lucia (27°52'0"S to 28°24'0"S and 32°21'0"E to 32°34'0"E) is located in the north-eastern corner of South Africa in the KwaZulu-Natal province and is a large (~ 300 to 350 km^2^) estuarine lake system comprising three interconnected shallow lakes (South Lake, North Lake and False Bay) that are joined to the Indian Ocean via a 21 km channel known as the Narrows (Fig. 1). Aquatic beetle samples were collected from a variety of freshwater habitats on the coastal plain surrounding the main expanse of Lake St Lucia, and from the vegetated margins of the estuarine lake body itself. Three collection trips were undertaken: November 2013 (19^th^–30^th^); July 2014 (23^rd^–24^th^); and January-February 2015 (31^st^ January – 6^th^ February). In total, 32 sites were sampled over the course of the three collection trips, encompassing a diverse array of habitats (Fig. 1).

Six waterbody types were sampled (following the classification of Ollis et al. 2015): depression wetlands (both isolated and non-isolated, n = 16); valley bottom wetlands (both channelled and un-channelled, n = 8); rivers (both in-channel and riparian habitats, n = 4); a wetland flat; a seepage wetland; and the estuarine lake body itself. Most of these waterbodies were extensively vegetated and some of the smaller depression and valley bottom wetlands were temporary. The locations sampled, their habitat classification and dates of sampling are summarised in Table 1.

**Table 1.** Geographic position and classification of the waterbodies sampled during this study. Sampling took place during the three collecting trips to Lake St Lucia during November 2013, July 2014 and January/February 2015. Classification (wetland type) follows the hydrogeomorphic (HGM) approach of Ollis et al. (2015). WS – Western Shores; ES – Eastern Shores; FB – False Bay. Table reproduced with permission from Perissinotto et al. (2016).

Site	GPS (D°M'S")		Wetland type	Region	Nov 2013	Jul 2014	Jan/ Feb 2015
1	28°20'53.33"S	32°23'38.42"E	River (pool)	WS		×	×
2	28°20'54.23"S	32°22'59.68"E	Depression	WS		×	
3	28°21'10.77"S	32°23'7.88"E	Channelled valley bottom	WS		×	
4	28°21'7.52"S	32°23'24.04"E	Channelled valley bottom	WS		×	
5	28°17'55.76"S	32°23'10.62"E	River (riparian zone)	WS		×	
6	28°15'26.06"S	32°23'36.51"E	Depression	WS	×	×	×
7	28°15'11.10"S	32°23'39.95"E	Depression	WS	×	×	×
8	28°12'25.44"S	32°24'22.97"E	Depression (artificial)	WS		×	
9	28°15'19.19"S	32°23'38.53"E	Depression	WS		×	
10	28°17'19.08"S	32°23'16.53"E	Depression	WS		×	
11	28°18'31.52"S	32°26'54.54"E	Un-channelled valley bottom	ES		×	
12	28°17'00.81"S	32°27'43.78"E	Depression	ES		×	
13	28°16'6.26"S	32°28'00.02"E	Depression	ES		×	×
14	28°16'10.26"S	32°27'35.43"E	Depression	ES		×	×
15	28°18'25.29"S	32°26'59.88"E	Un-channelled valley bottom	ES		×	
16	28°14'15.05"S	32°24'32.30"E	Depression	WS			×
17	28°15'1.00"S	32°24'9.85"E	Channelled valley bottom	WS			×
18	28°17'44.59"S	32°22'58.49"E	Flat	WS			×
19	28°07'10.99"S	32°31'8.98"E	Un-channelled valley bottom	ES			×
20	28°12'21.75"S	32°29'27.07"E	River (main channel)	ES			×
21	28°20'59.06"S	32°25'50.76"E	Depression	ES			×
22	28°18'59.92"S	32°26'10.64"E	Depression	ES			×
23	28°20'7.84"S	32°26'10.36"E	Depression	ES			×
24	28°22'44.46"S	32°25'20.13"E	River (connected to estuary)	ES			×
25	28°21'59.12"S	32°25'42.10"E	Depression	ES			×
26	27°58'32.33"S	32°21'51.14"E	Depression	FB			×
27	27°57'31.50"S	32°21'41.82"E	Depression	FB	×		×
28	27°58'25.01"S	32°22'16.02"E	Channelled valley bottom	FB			×
29	28°00'51.44"S	32°21'54.93"E	Channelled valley bottom	FB	×		×
30	28°00'47.95"S	32°22'00.92"E	Estuarine lake	FB	×		×
31	28°02'9.17"S	32°21'42.78"E	Estuarine lake shore (light trap)	FB	×	×	×
32	28°13'14.56"S	32°29'12.45"E	Seep	ES			×

### Field sampling protocol

Beetle collection efforts primarily involved the use of a long-handled square-framed sweep net (30 cm mouth and 1 mm mesh), following a sweep protocol similar to that of Bilton et al. (2006) and Bird et al. (2013). Sampling effort was concentrated in submerged vegetation and at the shore margins. A semi-quantitative approach was incorporated, whereby approximately 20 sweeps from the water surface to bottom substrate and back to the surface were performed at each waterbody. Visual searches for beetles at the shore margin and light trapping were also conducted to complement the array of taxa collected with the sweep net. The light trap consisted of a 4×3 m white sheet that was hung vertically below a fluorescent mercury vapour lamp (Radiant 250 W) and was deployed on all three survey trips at False Bay, adjacent to the lake shore (site 31, Table 1), during the evening (20:00-05:00 hrs). Aquatic coleopteran specimens were retrieved from the light sheet by hand. All beetle specimens collected during the November 2013 and July 2014 surveys were killed using ethyl acetate vapour and preserved in 5% formalin solution. Specimens collected during January-February 2015 were killed in the same way and preserved in 70% ethanol.

A range of *in situ* physico-chemical parameters were measured at each site. Salinity, temperature, pH, dissolved oxygen and turbidity were recorded using a YSI 6600-V2 multi-system probe. Due to technical problems, physico-chemical measurements were not taken during November 2013.

### Historical data and other collections

Aquatic Polyphaga collections housed in the major South African museums, namely the Iziko South African Museum (ISAM, Cape Town), the Ditsong National Museum of Natural History (DNMNH, Pretoria; formerly the Transvaal Museum) and the South African National Collection of Insects (SANC, Pretoria), were databased by the respective curators to add historical records to this study. Further data on species collected during previous surveys in the St Lucia area were obtained from Day et al. (1954), Millard and Broekhuysen (1970), Vrdoljak (2004) and Perkins (2014). Records of specimens collected by the authors of the current study during preliminary investigations in the area carried out between 2008 and 2012 were also included. Most of this historical material has, however, not been examined by taxonomic specialists, except for the 2008-2012 collections. Identifications should therefore be considered with caution.

### Identification and illustrations

Species identification was undertaken with reference to museum material and the most recent literature available on the specific taxa. Characteristics of male genitalia were generally used as the key criterion for species identification and separation. Digital photographs of the dorsal habitus of each species were taken using a EOS 600D digital camera fitted to a Sigma 50mm f/2.8 EX DG macro lens for larger specimens (≥ 1.5 cm) and a Leica Z6 APO for smaller specimens (< 1.5 cm). Image stacks were produced by hand, and combined using Zerene Stacker software (www.zerenesystems.com). To facilitate future identification and monitoring exercises, an annotated and illustrated list was compiled of all species identified in the preliminary collections of 2008-2012 and during the three dedicated surveys conducted in November 2013, July 2014 and January/February 2015 (Appendix 1).

### Statistical analysis

Multivariate techniques were used to analyse spatial trends in the composition of polyphagan beetle assemblages at St Lucia. Beetle data were converted to presence-absence and assemblage similarity amongst sites was analysed using the Bray-Curtis coefficient. Non-metric multidimensional scaling (MDS) was used to depict beetle assemblages at St Lucia on a two-dimensional plot. Differences in beetle assemblage composition across the regions of St Lucia (Eastern Shores, Western Shores and False Bay) and waterbody types (excluding seeps and flats as only one of each was sampled) were tested using non-parametric permutational MANOVA (PERMANOVA, Anderson 2001), using the Bray-Curtis coefficient for construction of the dissimilarity matrix. As with the multivariate data (assemblage composition), the univariate measure of species richness (number of species per waterbody) was also compared across both regions and waterbody types, this time using the non-parametric Kruskal-Wallis test. Linear regression was used to assess the relationship between the number of polyphagan taxa recorded per site and that recorded for the adephagans by Perissinotto et al. (2016).

Multidimensional scaling was performed using PRIMER v6 software (Clarke and Warwick 2001, Clarke and Gorley 2006). Non-parametric permutational MANOVA was performed using the PERMANOVA routine in the PERMANOVA+ add-on package (Anderson et al. 2008) to PRIMER v6 software. P-values for PERMANOVA models were tested using 9999 unrestricted permutations of the raw data. The Kruskal-Wallis tests on species richness and linear regression were performed using Statistica 12 software for Windows (Statsoft Inc. 2015). All tests were performed using an *a priori* significance level of α = 0.05.

## Results

The sites sampled during this survey reflect the relative abundance of the various waterbody types encountered on the St Lucia coastal plain, with groundwater-fed depressions and valley bottom wetlands predominating, although several small rivers, a wetland flat and a seep were also sampled, in addition to the estuarine lake itself. Freshwater wetlands around Lake St Lucia were mostly small (< 2 ha), shallow (< 1 m maximum depth) and extensively vegetated. Further details on the physico-chemistry of the waterbodies sampled at St Lucia are provided by Perissinotto et al. (2016).

### Polyphaga collected during the current survey

A total of 37 taxa of aquatic Polyphaga were collected during the three dedicated surveys of the current study (2013–2015), which are listed in Table 2 and illustrated in the checklist (see Appendix 1). The survey revealed a new species of Hydrophilidae, recently described as *Hydrobiomorpha perissinottoi* Bilton, 2016 (Bilton 2016). In addition, five species represent new records for the Republic of South Africa, four of which are hydrophilids (*Paracymus exiguus* Wooldridge, 1977; *Amphiops uhligi* Hebauer, 1995; *Hydrochara fulvofemorata* (Fairmaire, 1869) and *Laccobius uhligi* Gentili, 1995). The other species is a hydraenid (Aulachochthebius cf. continentalis (Orchymont, 1929)), which, whilst new to South Africa, has not been identified with certainty. This genus is currently being revised (Phil Perkins, *pers. comm.*). Of the 37 polyphagan taxa collected in this study, 27 were identified to species level. The remaining 10 taxa were assigned to the following (sub)genera, and could not be named reliably to species: *Hydrochus*; *Allocotocerus* Kraatz, 1883; Enochrus (Methydrus) Thomson, 1859; *Helochares* Mulsant, 1844; and *Coelostoma* Brullé, 1835. In the case of these genera, taxa were assigned to morphospecies, but further taxonomic work, including in some cases generic revisions, would be required to name species with confidence, but such taxonomic uncertainly does not affect our analyses. Hydrophilidae dominated the polyphagan beetle assemblages at St Lucia, being represented by 27 species. Relatively minor representation was afforded by the Hydrochidae (three species of *Hydrochus*); Spercheidae (two species, *Spercheus cerisyi* Guérin-Méneville, 1842 and *Spercheus senegalensis* Castelnau, 1832); Hydraenidae (four species, *Hydraena cooperi* Balfour-Browne, 1954, *Limnebius probus* Perkins, 2015, Aulachochthebius cf. continentalis and *Ochthebius andronicus* Orchymont, 1948); and Curculionidae (one species, Pseudobagous cf. longulus (Gyllenhal, 1836)).

**Table 2.** Polyphagan beetles collected from St Lucia during the course of this study. The sites are listed from which each taxon was collected on each of the three sampling trips. Site numbers 1 – 32 correspond to those listed in Table 1. The regions where each taxon occurred are also indicated: WS – Western Shores; ES – Eastern Shores; FB – False Bay. Species new to South Africa are shown in bold type. Classification of Hydrophilidae follows Short and Fikáček (2013).

Taxon	Sampling date			Region		
	Nov 2013	Jul 2014	Jan/Feb 2015	WS	ES	FB
Hydrochidae:						
*Hydrochus* sp. 1		2, 3, 5, 13, 14, 15	6, 7, 13, 14, 16, 17, 18, 21, 22, 23, 27, 32	×	×	×
*Hydrochus* sp. 2			6, 14, 17, 19, 27, 32	×	×	×
*Hydrochus* sp. 3			18	×		
Spercheidae:						
*Spercheus cerisyi* Guérin-Méneville, 1842			6, 7, 14, 16, 17, 18, 21, 27	×	×	×
*Spercheus senegalensis* Castelnau, 1832			6, 14, 17, 18, 20, 25, 27	×	×	×
Hydrophilidae:						
*Amphiops globus* Erichson, 1843			1, 20, 27	×	×	×
*Amphiops senegalensis* (Laporte, 1840)		15	1, 7, 14, 16, 22, 23, 25, 27	×	×	×
***Amphiops uhligi* Hebauer, 1995**			14		×	
*Allocotocerus* sp.	27	10	6, 14, 18, 23, 27, 29	×	×	×
*Berosus cuspidatus* Erichson, 1843			6, 7, 14, 18, 21, 22, 27, 28, 29, 31, 32	×	×	×
*Berosus viticollis* Boheman, 1851	7, 29, 30			×		×
*Regimbartia nilotica* (Sharp, 1903)	27		6, 14, 18, 21, 27, 29	×	×	×
*Regimbartia obsoleta* (Régimbart, 1906)			14, 18, 22, 27, 29	×	×	×
***Laccobius uhligi* Gentili, 1995**			32		×	
+ *Paracymus amplus* Wooldridge, 1977			21		×	
***Paracymus exiguus* Wooldridge, 1977**			7, 13, 18, 21, 29	×	×	×
*Paracymus pisanus* Balfour-Browne, 1954			7, 13, 14, 18, 21, 25, 27, 29, 32	×	×	×
*+*Hydrobiomorpha perissinottoi* Bilton, 2016			16, 18, 22, 29	×	×	×
*Hydrochara elliptica* (Fabricius, 1801)	31					×
***Hydrochara fulvofemorata* (Fairmaire, 1869)**	30		6, 16, 17, 26, 27, 29, 31	×		×
*Hydrophilus aculeatus* (Solier, 1834)	31	10	14, 31	×	×	×
*Sternolophus solieri* Laporte, 1840	30		14, 17, 18, 20, 22, 23, 24, 27, 31	×	×	×
Enochrus (Methydrus) sp.	30	1, 4, 5	7, 13, 14, 16, 17, 18, 19, 21, 27, 28, 29, 32	×	×	×
Chasmogenus cf. patrizii (Balfour-Browne, 1948)			14, 23, 27, 29		×	×
*Helochares dilutus* (Erichson, 1843)		10	6, 21, 24, 27, 28, 29, 31	×	×	×
*Helochares longipalpis* (Murray, 1859)		11	14, 16, 17, 22, 23, 27, 29, 31, 32	×	×	×
*Helochares* sp. 1		12	27		×	×
*Helochares* sp. 2		3, 4, 5, 15	1, 6, 7, 14, 17, 18, 19, 21, 23, 24, 25, 27, 29, 31	×	×	×
*Coelostoma* sp. 1			14, 20, 23, 27, 31, 32		×	×
*Coelostoma* sp. 2	29, 30	1, 10	18, 23, 24, 26, 27, 28, 31, 32	×	×	×
*Coelostoma* sp. 3			22, 23		×	
*Cercyon dieganus* Régimbart, 1903			1, 14, 16, 22, 23, 27, 28, 31	×	×	×
Hydraenidae:						
*Hydraena cooperi* Balfour-Browne, 1954			14, 17, 21, 22, 25, 29, 32	×	×	×
+ *Limnebius probus* Perkins, 2015			27, 29			×
**Aulachochthebius cf. continentalis (Orchymont, 1929)**			21, 27, 29, 32		×	×
*Ochthebius andronicus* Orchymont, 1948			21, 29		×	×
Curculionidae:						
Pseudobagous cf. longulus (Gyllenhal, 1836)		13, 14	29		×	×

+ Taxa known only from South Africa.

* New species, first found in this study.

Polyphagan beetles were generally widespread across a number of waterbodies, with 21 of the 37 species being collected from five or more sites (Table 2). The three most widespread species were *Helochares* sp. 2, collected from 18 waterbodies, and *Hydrochus* sp. 1 and Enochrus (Methydrus) sp., both collected from 16 waterbodies. Only five species (*Hydrochus* sp. 3, *Paracymus amplus*, *Amphiops uhligi*, *Hydrochara elliptica* and *Laccobius uhligi*) were recorded from a single waterbody (Table 2).

### Historical records

Polyphagan taxa collected from St Lucia and surroundings prior to the current survey are listed in Table 3. A total of 49 taxa were previously recorded from the region, but of these, only 19 represent species-level records. Nineteen unpublished museum records were found from our extensive search of museum collection records across South Africa (including material stored by the authors at UKZN). Of these, 15 are species-level records, 10 of which were also recorded during the collections of the current study (2013-2015: indicated by an asterisk in Table 3). Thirty-one aquatic polyphagan taxa have been reported for St Lucia from previously published studies of the system (unpublished M.Sc. thesis in the case of Vrdoljak 2004), although only five of these are species-level records (three of which were also recorded in the current study), reflecting the ecological rather than taxonomic focus of these studies. Although Heteroceridae and Scirtidae are listed in Table 3, members of these families were not recorded in the collections of the current study.

**Table 3.** Aquatic polyphagan beetles previously recorded from the Lake St Lucia system and surrounding waterbodies. Literature sources are indicated by letters as follows: (a) Day et al. (1954); (b) Millard and Broekhuysen (1970); (c) Vrdoljak (2004); and (d) Perkins (2014). Museum and national collection material is as follows: SANC – South African National Collection of Insects; ISAM – Iziko South African Museum; DNMNH – Ditsong National Museum of Natural History. Locations referred to are: FWW – fresh water wetlands on the Eastern Shores of Lake St Lucia; FB – False Bay; SL – St Lucia (lake body and immediate surrounds); KB – Kosi Bay; D – Dukuduku; DF – Dukuduku forest; DP – Dukandlovu Pan (site 29 in the current study). Also included here are records based on ad hoc collections undertaken by the authors during the period 2008-2012 (deposited at the University of KwaZulu-Natal and listed as UKZN).

Family	Genus	Species	Publication	Years recorded	Location
Hydrochidae	*Hydrochus* Leach, 1817	*Hydrochus* spp. 1–4	(c)	2002/2003	FWW
Spercheidae	*Spercheus* Illiger, 1798	Spercheus cerisyi*	SANC	Not specified	D
		Spercheus senegalensis*	SANC	Not specified	SL, D
Hydrophilidae	*Allocotocerus* Kraatz, 1883	*Allocotocerus* sp.	(c)	2002/2003	FWW
	*Amphiops* Erichson, 1843	Amphiops senegalensis*	(c)	2002/2003	FWW
		*Amphiops* spp. 1–2	(c)	2002/2003	FWW
	*Anacaena* Thomson, 1859	*Anacaena* sp.	(c)	2002/2003	FWW
	*Berosus* Leach, 1817	Berosus cuspidatus*	(a), (b)	1948	FB
			DNMNH	1960	SL
		*Berosus* spp. 1–3	(c)	2002/2003	FWW
	*Coelostoma* Brullé, 1835	*Coelostoma rufitarse* (Boheman, 1851)	(a), (b)	1948	FB
		*Coelostoma* spp. 1–4	(c)	2002/2003	FWW
	*Dactylosternum* Wollaston, 1854	*Dactylosternum* sp.	(c)	2002/2003	FWW
	*Enochrus* Thomson, 1859	*Enochrus* spp. 1–4	(c)	2002/2003	FWW
	*Helochares* Mulsant, 1844	Helochares dilutus*	SANC	Not specified	SL, D
		Helochares longipalpis*	SANC	Not specified	SL, D
		*Helochares* spp. 1–4	(c)	2002/2003	FWW
	*Hydrochara* Berthold, 1827	Hydrochara elliptica*	UKZN	2012	DP
	*Regimbartia* Zaitzev, 1908	*Regimbartia* sp.	(c)	2002/2003	FWW
Hydrophilidae	*Sternolophus* Solier, 1834	Sternolophus solieri*	UKZN	2008	FB
		*Sternolophus angolensis* (Erichson, 1843)	(c)	2002/2003	FWW
		*Sternolophus* sp.	(c)	2002/2003	FWW
	*Hydrophilus* Geoffroy, 1762	Hydrophilus aculeatus*	SANC	Not specified	D, SL
Hydrophilidae	*Hydrophilus* Geoffroy, 1762	*Hydrophilus senegalensis* (Percheron 1835)	UKZN	2012	FB
		*Hydrophilus* sp.	ISAM	1988	KB
	*Chasmogenus* Sharp, 1882	*Chasmogenus lycetus* (d’Orchymont, 1939)	SANC	Not specified	SL
		Chasmogenus patrizii*	SANC	Not specified	SL
Hydraenidae	*Hydraena* Kugelann, 1794	Hydraena cooperi*	(d)	1997	
Scirtidae	*Cyphon* Paykull, 1799	*Cyphon* sp.	DNMNH	Not specified	SL, DF, KB
	*Scirtes* Illiger, 1807	*Scirtes* sp.	DNMNH	Not specified	SL, KB
	*Ora* Clark, 1865	*Ora* sp.	DNMNH	Not specified	SL
Heteroceridae	*Augyles* Schiödte, 1866	*Augyles pallens* (Charpentier, 1965)	SANC	Not specified	D
	*Heterocerus* Fabricius, 1792	*Heterocerus atroincertus* Charpentier, 1965	SANC	Not specified	SL
		*Heterocerus thebaicus australis* Charpentier, 1965	SANC	Not specified	D
Curculionidae	*Pseudobagous* Sharp, 1917	Pseudobagous longulus*	SANC	Not specified	D

* Also recorded during the dedicated surveys of 2013–2015.

### Patterns of assemblage composition

**Figure 2.**
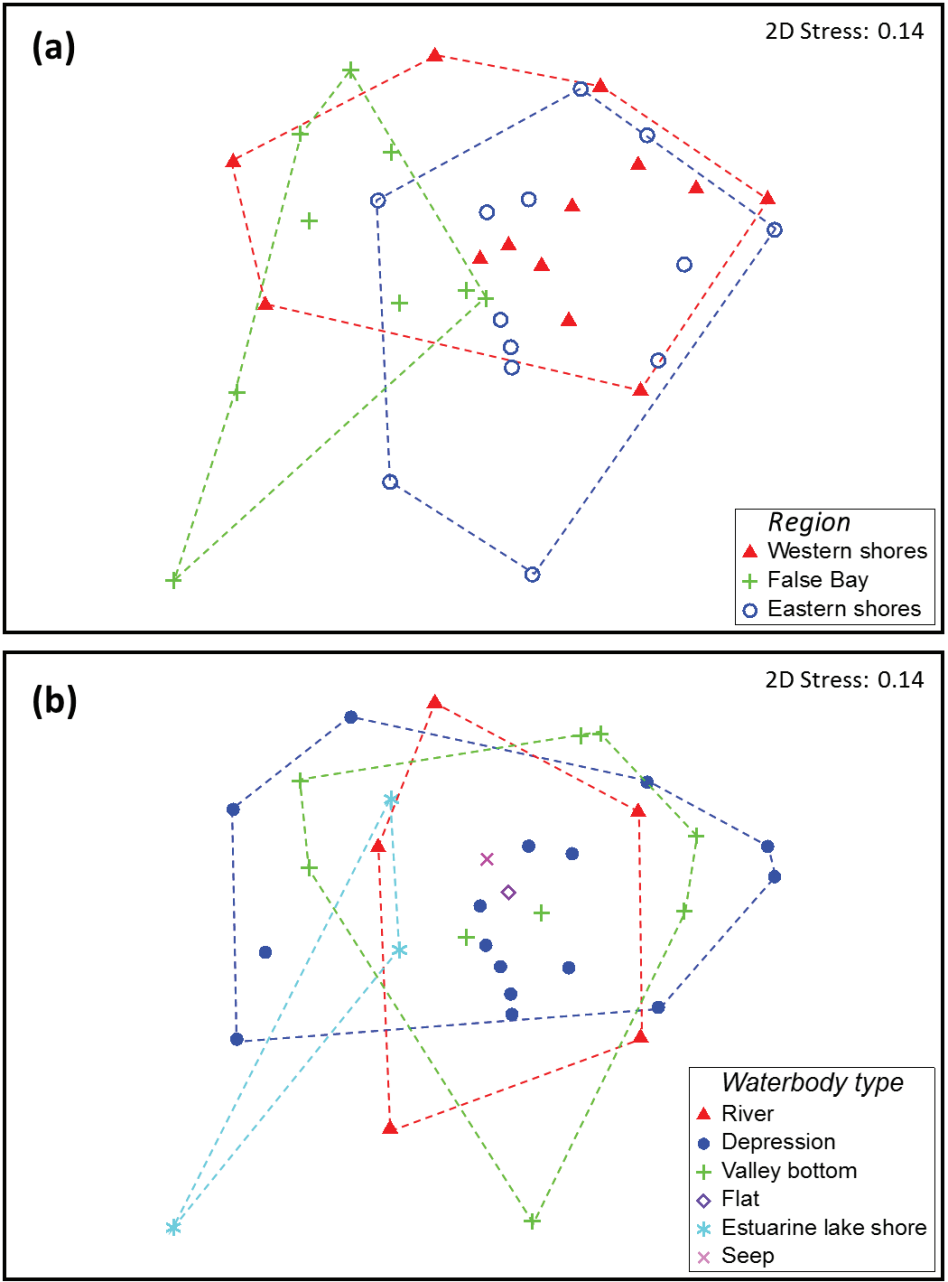
Multidimensional scaling (MDS) plot depicting the similarity of sites sampled in this study in terms of beetle assemblage composition. Symbols on the plot have been coded in terms of (**a**) region and (**b**) waterbody type. Convex hulls (dashed lines) have been overlaid on each plot to clarify groupings according to region/waterbody type.

The composition of polyphagan beetle assemblages was similar between the Western and Eastern Shores of St Lucia, as reflected by the high degree of overlap of sites from these two regions in the MDS plot (Fig. 2a). Assemblages from False Bay show some distinction from those of the other two regions, with sites generally placed toward the left of the plot in Fig. 2a. The PERMANOVA results (Table 4) confirm these patterns, reporting a significant overall difference in assemblage composition of sites across the three regions sampled, with post hoc pairwise tests indicating that the overall difference was driven by the distinctness of the False Bay sites (Table 4a). The different waterbody types at St Lucia did not appear to harbour distinct assemblages of polyphagan beetles, with sites from the different waterbody types overlapping widely in the MDS plot (Fig. 2b). This overlap is confirmed by the PERMANOVA results, reporting no significant difference in assemblage composition across wetland types (Table 4b).

**Table 4.** Non-parametric permutational MANOVA (PERMANOVA) results for models comparing beetle assemblage composition. Assemblage composition at St Lucia was compared across (a) regions, and (b) waterbody types. The multivariate models tested for differences between group centroids in Bray-Curtis dissimilarity space. Pairwise comparisons are reported in the case of (a), where overall test results were significant. WS – Western Shores; FB – False Bay; ES – Eastern Shores.

(a)						*Post hoc* pairwise comparisons		
Source	df	SS	MS	F	P	Groups	t	P
Region	2	13006	6502.9	1.9978	0.012*	WS, FB	1.5753	0.019*
Residual	30	100910	3255.1			WS, ES	0.85389	0.689
Total	32	113910				FB, ES	1.7283	0.002*
**(b)**								
**Source**	**df**	**SS**	**MS**	**F**	**P**			
Waterbody type	3	13102	4367.4	1.2997	0.144			
Residual	29	100810	3360.4					
Total	32	113910						

* Significant P values at α = 0.05.

### Species richness patterns

**Figure 3.**
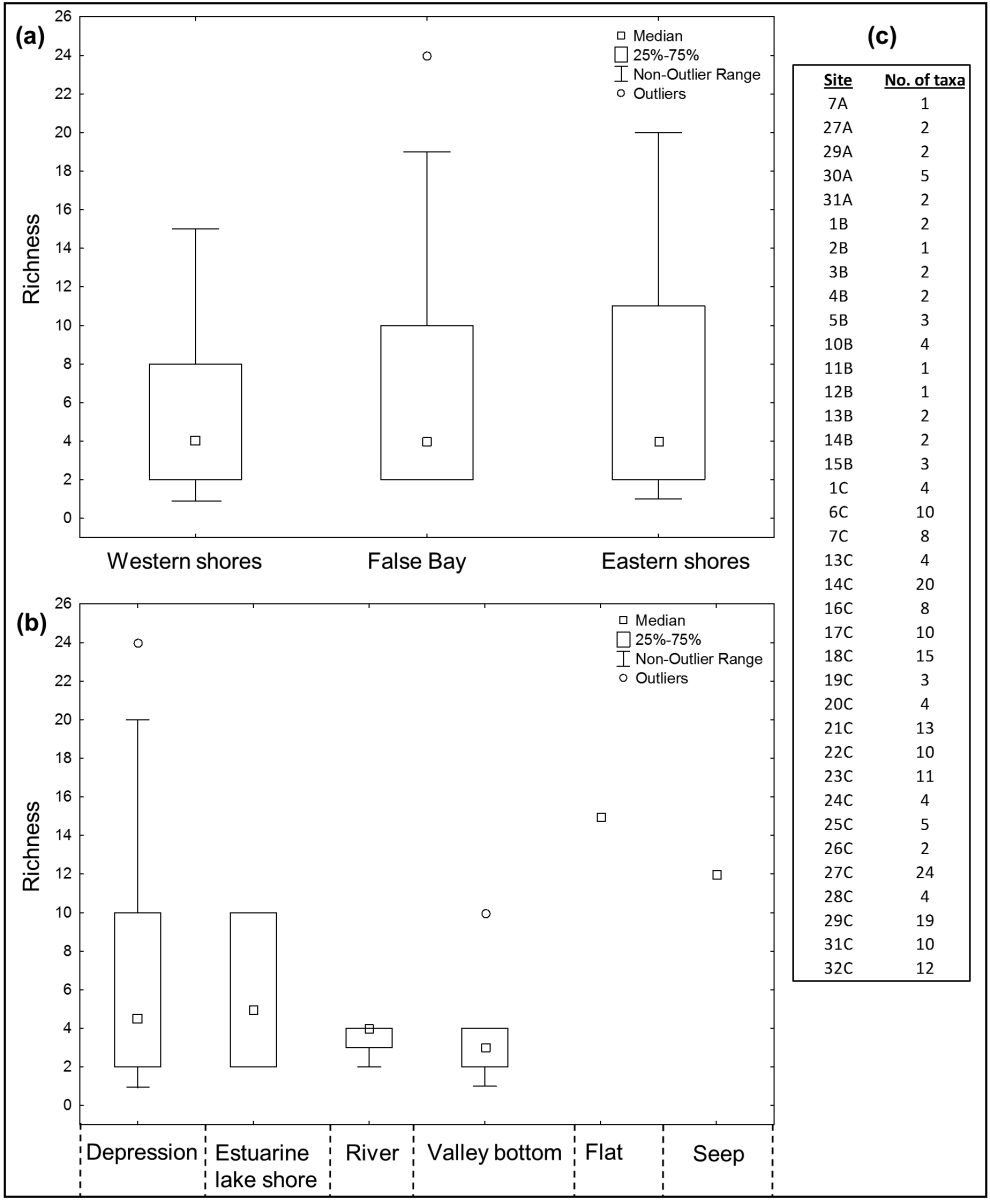
Box-plots comparing the median and spread of species richness (number of polyphagan taxa per site) among (a) regions and (b) waterbody types at St Lucia during the sampling period 2013–2015. The data representing number of taxa per site are also reported (c). Site numbers in (c) are coded as A (first survey–November 2013), B (second survey–July 2014) or C (third survey–January/February 2015). Kruskal-Wallis tests indicated that species richness did not vary significantly among regions (KW-H_2, 37_ = 0.9006, p = 0.6374) or waterbody types (KW-H_5, 37_ = 4.2675, p = 0.5116).

**Figure 4.**
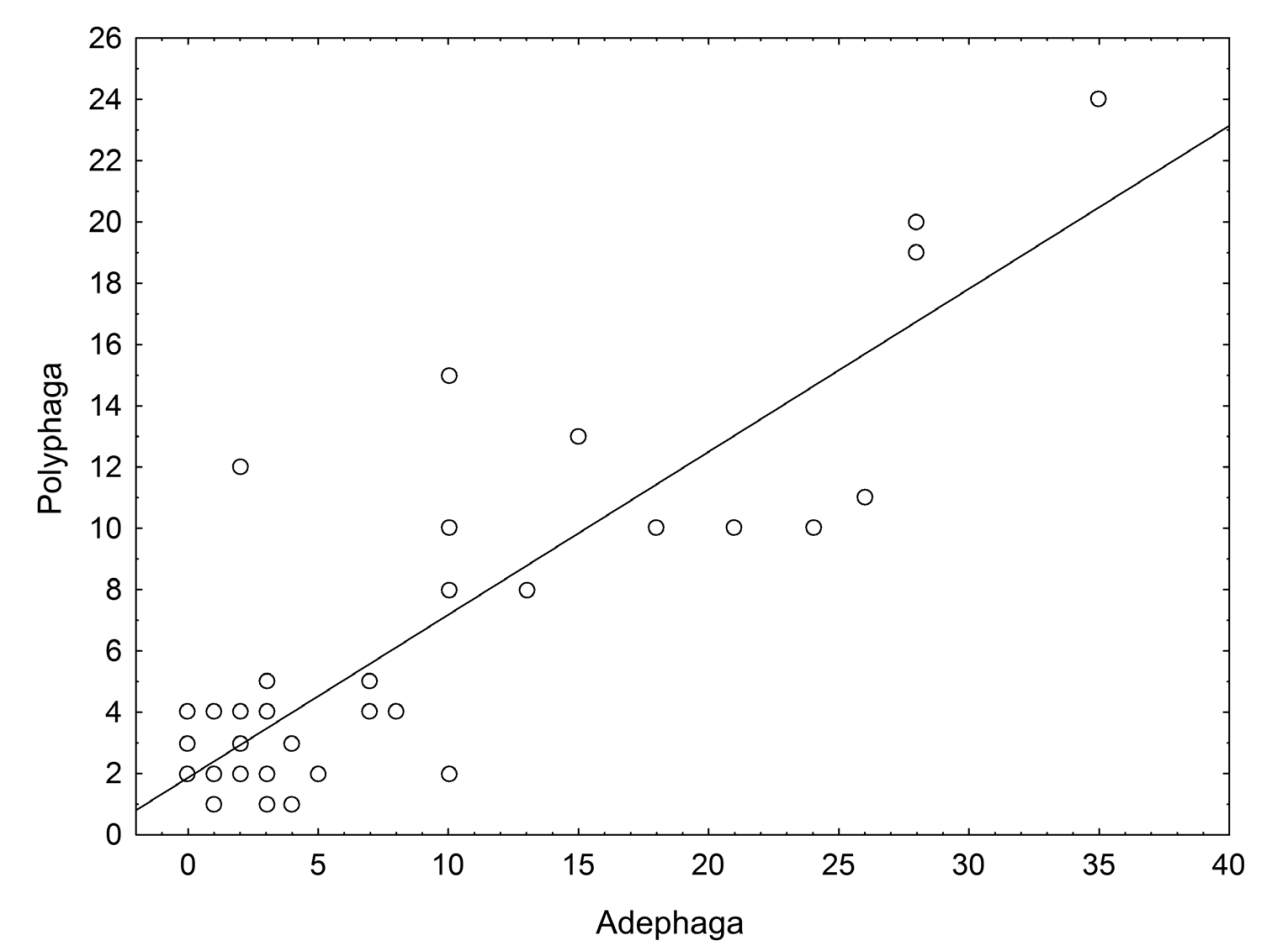
Scatterplot depicting the positive linear relationship (r = 0.8605, P < 0.001) between the number of taxa per site for Polyphaga (sampled in the current study) and Adephaga (concurrently sampled by Perissinotto et al. 2016).

Kruskal-Wallis tests showed that polyphagan beetle richness did not differ significantly between the three regions of St Lucia (KW-H_2, 37_ = 0.9006, p = 0.6374) or between waterbody types (KW-H_5, 37_ = 4.2675, p = 0.5116). Mean richness across all sites and sampling trips was 6.4±5.9 (SD) species per site, the high standard deviation reflecting large variation in the number of species recorded per site. The boxplots in Fig. 3 provide a visual depiction of the median and range of richness values across sites, grouped firstly by region (Fig. 3a) and secondly by waterbody type (Fig. 3b). Although median richness per site was low (~ 4 species) in each of the three regions of St Lucia, the distribution was skewed; the non-outlier ranges of all three regions including sites with 15 or more species (Fig. 3a). In terms of waterbody types, the boxplot (Fig. 3b) does not reveal any clear pattern of differences among groups. ‘Wetland flat’ and ‘seep’ categories were each represented by only a single site sampled on one occasion (January/February 2015) and were both relatively rich in taxa (15 and 12 species). Four sites yielded only a single species (sites 7A, 2B, 11B and 12B; Fig. 3c). The three richest samples were all collected during the mid-summer wet season sampling trip in January/February 2015, from a depression wetland at False Bay (site 27C with 24 species), a depression wetland on the Eastern Shores (site 14C with 20 species) and a valley bottom wetland at False Bay (site 29C with 19 species - Fig. 3c). Polyphagan species richness per site was highly correlated (r = 0.8605, P < 0.001, Fig. 4) with the richness of adephagans sampled concurrently at the same sites (Perissinotto et al. 2016).

## Discussion

The dedicated surveys of the St Lucia coastal plain between 2013 and 2015 have revealed 37 aquatic polyphagan species, which predominantly reside in the small freshwater wetlands surrounding the main lake body. Given that ca. 360 species of aquatic Polyphaga have been listed for southern Africa (Stals and de Moor 2007), the St Lucia system houses at least 10% of the aquatic polyphagan fauna of this biodiverse region. If historical records are taken into account and the seven species-level museum records (Table 3) are added to the 37 species collected during the current study, then St Lucia apparently supports at least 12% of regional diversity.

The number of Polyphaga collected at St Lucia represents approximately half the richness of hydradephagan beetles (68 taxa) reported from the same set of waterbodies by Perissinotto et al. (2016). A greater richness of the Adephaga over Polyphaga in aquatic systems has been reported elsewhere. Apenborn (2013) reported 122 species of water beetles from eight weeks of collecting effort at the Panguana research station in lowland rainforest of central Peru (Hendrich et al. 2015). Of these, around 40 belonged to the Polyphaga and 80 to the Adephaga; a ratio of ~1:2 (Polyphaga: Adephaga) as in the current study. However, this is not always the case and the ratio appears to vary regionally. For instance, Lake Najun (approximately 100 km^2^) and its immediate tributaries in the Philippines produced 49 coleopteran species, of which 38 belonged to the Polyphaga, with only 10 Adephaga (Freitag and Pangantihon 2010). In a comprehensive checklist of aquatic beetle diversity of the Iberian Peninsula in the Mediterranean region, Ribera et al. (1998) reported 622 aquatic beetle species, however, largely due to the high regional richness of Hydraenidae (138 species), the total number of Polyphaga was 401 species, considerably outnumbering the Adephaga at 198 species. Chaco National Park and El Cachapé Wildlife Refuge in the humid Chaco Province of northern Argentina, which similarly to St Lucia is also a sub-tropical lowland plain area, albeit non-coastal, yielded 122 species, of which approximately half (60 species) belonged to the Polyphaga and the remainder were adephagans (Libonatti et al. 2013). In terms of richness, the samples were dominated by Hydrophilidae (43 species), which here even outnumbered Dytiscidae (37 species). At St Lucia Dytiscidae make up a much larger component of the fauna than Hydrophilidae, with 52 dytiscid species (Perissinotto et al. 2016) in comparison to the 27 hydrophilid species reported from the same waterbodies in the current study. This ratio is roughly in line with that reported for the Afrotropical region as a whole, given that approximately 1,060 dytiscid species have been described thus far for the region in comparison to ca. 450 hydrophilids (Jäch and Balke 2008). In their global assessment of aquatic coleopteran species diversity, Jäch and Balke (2008) report a total of approximately 7,130 polyphagan species, in comparison to 5,126 Adephaga species, a ratio of ~1.4:1 in favour of Polyphaga. In the Afrotropical region the same authors report 1,400 species of Adephaga versus 1,200 of Polyphaga (Jäch and Balke 2008), a ratio of ~1.2:1 in favour of Adephaga. One reason for the relatively low numbers of polyphagan species compared to Adephaga in the current study is the complete absence of fast running waters from this lowland region. Such habitats support large numbers of species in families such as Hydraenidae and Elmidae, including in adjacent parts of South Africa, but such beetles were entirely absent from the areas sampled in iSimangaliso Park due to the lack of suitable habitats.

The St Lucia Polyphaga were generally dominated by widespread Afrotropical taxa, with only two endemic South African species being recorded (*Paracymus amplus* Wooldridge, 1977 and *Limnebius probus* Perkins, 2015). A similar pattern (dominance of widespread taxa) is apparent at St Lucia for other invertebrate groups such as the hydradephagan beetles (Perissinotto et al. 2016), gastropods (Perissinotto et al. 2014), bivalves (Nel et al. 2012) and odonates (Hart et al. 2014). The pattern of high richness and low endemism for polyphagan water beetles at St Lucia adds further evidence to the notion that invertebrate endemism decreases, whilst diversity increases, from the south-west to the north-east of South Africa (Stals and de Moor 2007, Perissinotto et al. 2016). A new species of Hydrophilidae, *Hydrobiomorpha perissinottoi*, was discovered and described from the collections of the current study (see Bilton 2016). This species was collected in good numbers in a variety of peripheral wetlands within the St Lucia system, although it is likely that its distribution extends throughout the broader region of Maputaland and beyond. Five species recorded in this survey are new to South Africa (Table 2), highlighting the poor state of knowledge of aquatic beetle distribution patterns in the region (see also Stals and de Moor 2007). Our survey highlights the need for further taxonomic work on some genera in the region, including *Hydrochus*, *Enochrus*, *Helochares* and *Coelostoma*, for which reliable species-level determinations are currently difficult or impossible.

In terms of the distribution of species within St Lucia, only five taxa (*Berosus viticollis* Boheman, 1851, Enochrus (Methydrus) sp., *Hydrochara fulvofemorata*, *Sternolophus solieri* Laporte, 1840 and *Coelostoma* sp. 2) were recorded from the margins of the lake body itself, and most taxa were instead found only in surrounding freshwater wetlands. Twelve taxa were taken with a light trap set up near the lake shore (sites 31A and 31C in November 2013 and February 2015 respectively), which captured flying adults that most likely were dispersing, perhaps from the nearby lake body. Polyphagan beetles formed a relatively distinct assemblage at False Bay, whilst the Western and Eastern Shores harbored very similar assemblages (Fig. 2a, Table 4). This same pattern was reported by Perissinotto et al. (2016) for hydradephagan beetle assemblages at St Lucia. Whilst the Eastern and Western Shores sites were generally on grassy sunlit plains, the False Bay sites occurred in dense dry woodland and were often heavily shaded, with lower resultant water temperatures (see Perissinotto et al. 2016). Although False Bay may have been distinctive in terms of its assemblage composition, the various regions of St Lucia appear to support approximately even numbers of polyphagan taxa and no region in particular was significantly elevated in terms of its species richness (Figure 3a), a finding similarly reported for the Hydradephaga of St Lucia by Perissinotto et al. (2016).

Polyphagan beetle assemblage composition did not differ between waterbody types (Fig. 2b, Table 4b), suggesting that the distribution of the species sampled, at least in the case of adults, is not affected by wetland type. Species richness also did not differ across waterbody types (Fig. 3b), further suggesting that beetles occur across the wide range of freshwater wetland type at St Lucia. That said, the two most species-rich sites sampled (20 and 24 species in sites 14C and 27C respectively) were both depression wetlands. A similar, but more pronounced pattern was reported for the Hydradephaga by Perissinotto et al. (2016), who showed that 5 of the 6 most speciose St Lucia sites were temporary depression wetlands. Polyphagan richness per site was strongly correlated with hydradephagan richness (Fig. 4) and the three most speciose sites in the current study (sites 27C, 14C and 29C) were also the most speciose for Hydradephaga. In contrast to the high beetle diversity recorded from small temporary freshwater wetlands in this study and by Perissinotto et al. (2016), other aquatic invertebrate assemblages at St Lucia have been found to be more diverse in the permanent fresh waterbodies (e.g. Hart et al. 2014) or in the estuarine lake body itself (e.g. Nel et al. 2012, Peer et al. 2014, Perissinotto et al. 2014).

## Conclusions

The majority of prior aquatic research within the iSimangaliso Wetland Park has focused on the estuarine lake itself, rather than the surrounding freshwater wetlands. Our study adds evidence in addition to that of Perissinotto et al. (2016) (hydradephagan beetles) and Hart et al. (2014) (odonates) that a high biodiversity is supported by the lesser-known freshwater systems of the park, emphasizing the importance of this UNESCO World Heritage Site as a biodiversity hotspot worthy of long-term conservation efforts. Although the park itself enjoys World Heritage status, much of the St Lucia catchment has become degraded by intensive land-use practices such as commercial plantations and agriculture. These practices have resulted in a drastically reduced input of freshwater to the lake system (Perissinotto et al. 2013) that, together with a prolonged drought between 2002 and 2010, caused severe hypersalinity in the estuarine lake and a drastic loss of biodiversity (Whitfield and Taylor 2009, Carrasco and Perissinotto 2012, Bird et al. 2016). Sampling in the current study was undertaken during a relatively wet period for the system, following the almost decade-long drought and hence most of the temporary wetlands on the coastal plain were flooded. Models of future climate conditions for the region predict increased variability in rainfall and an increase in extreme climatic events (e.g. drought or flood) linked to global climate change (Shongwe et al. 2009, Davis 2011, Dallas and Rivers-Moore 2014). Further study is thus warranted into the potential effects of a changing climate and intensifying catchment land use on the invertebrate assemblages that inhabit the park’s freshwater wetlands. Our results, taken together with those of Perissinotto et al. (2016), indicate that beetle biodiversity in freshwater ecosystems of the iSimangaliso Wetland Park is relatively high in a southern African context. However, to what degree this is an artefact of the generally poor state of knowledge of southern African water beetles can be better revealed by conducting comparably rigorous studies in other freshwater habitats of the region.

## Supplementary Material

XML Treatment for Hydrochus sp. 1

XML Treatment for Hydrochus sp. 2

XML Treatment for Hydrochus sp. 3

XML Treatment for Spercheus cerisyi

XML Treatment for Spercheus senegalensis

XML Treatment for Amphiops globus

XML Treatment for Amphiops senegalensis

XML Treatment for Amphiops uhligi

XML Treatment for Allocotocerus sp.

XML Treatment for Berosus cuspidatus

XML Treatment for Berosus viticollis

XML Treatment for Regimbartia nilotica

XML Treatment for Regimbartia obsoleta

XML Treatment for Laccobius uhligi

XML Treatment for Paracymus amplus

XML Treatment for Paracymus exiguus

XML Treatment for Paracymus pisanus

XML Treatment for Hydrobiomorpha perissinottoi

XML Treatment for Hydrochara elliptica

XML Treatment for Hydrochara fulvofemorata

XML Treatment for Hydrophilus aculeatus

XML Treatment for Sternolophus solieri

XML Treatment for Enochrus (Methydrus) sp.

XML Treatment for Chasmogenus cf. patrizii

XML Treatment for Helochares dilutus

XML Treatment for Helochares longipalpis

XML Treatment for Helochares sp. 1

XML Treatment for Helochares sp. 2

XML Treatment for Coelostoma sp. 1

XML Treatment for Coelostoma sp. 2

XML Treatment for Coelostoma sp. 3

XML Treatment for Cercyon dieganus

XML Treatment for Hydraena cooperi

XML Treatment for Limnebius probus

XML Treatment for Aulachochthebius cf. continentalis

XML Treatment for Ochthebius andronicus

XML Treatment for Pseudobagous cf. longulus

## Figures and Tables

**Figure 5.**
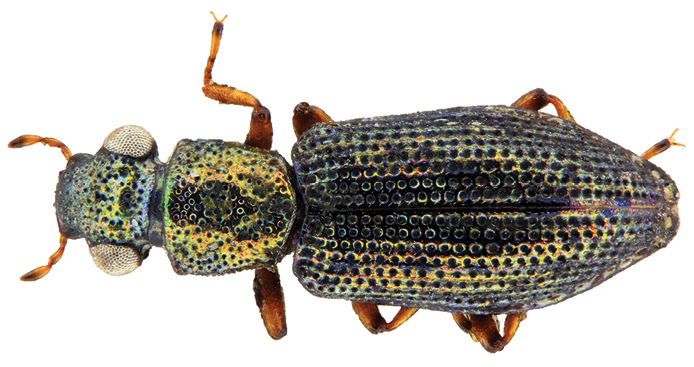
*Hydrochus* sp. 1 2.5 mm, iSimangaliso Wetland Park, Catalina Bay (site 32), February 2015 DT Bilton, MS Bird & R Perissinotto leg.

**Figure 6.**
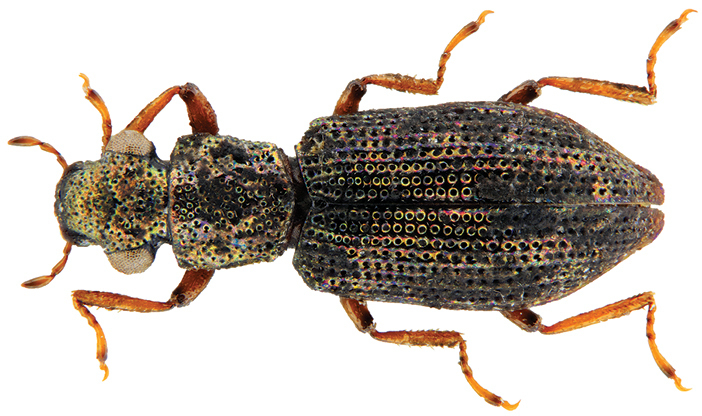
*Hydrochus* sp. 2 2.6 mm, iSimangaliso Wetland Park, Catalina Bay (site 32), February 2015 DT Bilton, MS Bird & R Perissinotto leg.

**Figure 7.**
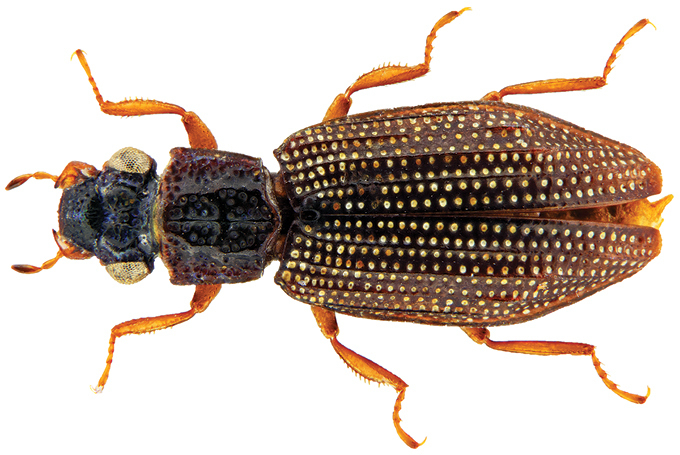
*Hydrochus* sp. 3 2.4 mm, iSimangaliso Wetland Park, Western Shores (site 18), February 2015 DT Bilton, MS Bird & R Perissinotto leg.

**Figure 8.**
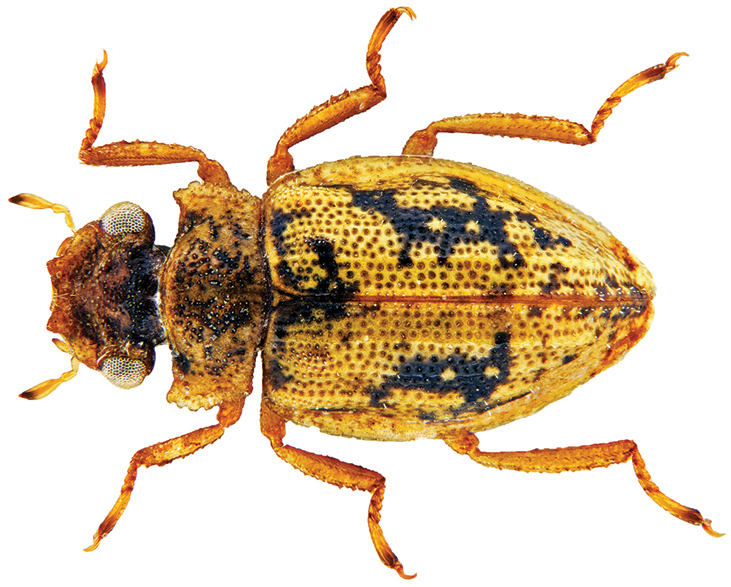
*Spercheus cerisyi* Guérin-Méneville, 1842 2.4 mm, iSimangaliso Wetland Park, Eastern Shores (site 14), February 2015 DT Bilton, MS Bird & R Perissinotto leg.

**Figure 9.**
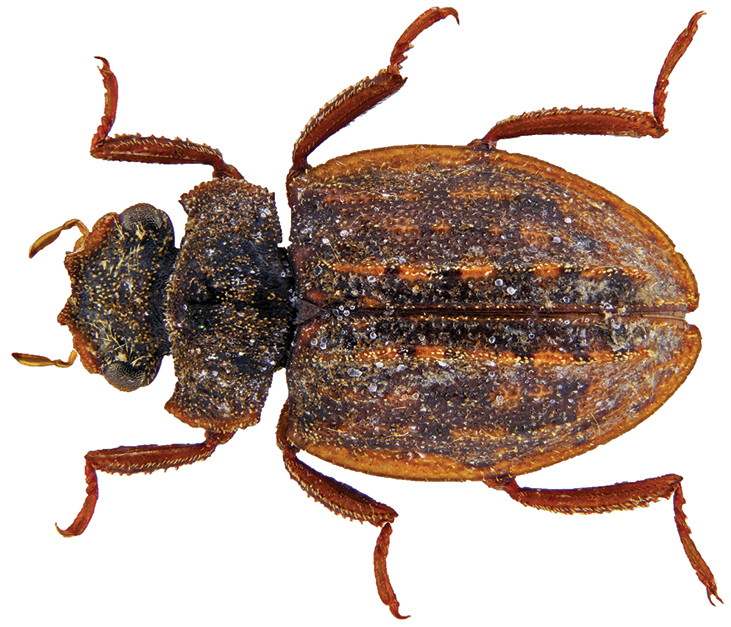
*Spercheus senegalensis* Castelnau, 1832 4.5 mm, iSimangaliso Wetland Park, False Bay (site 29), February 2015 DT Bilton, MS Bird & R Perissinotto leg.

**Figure 10.**
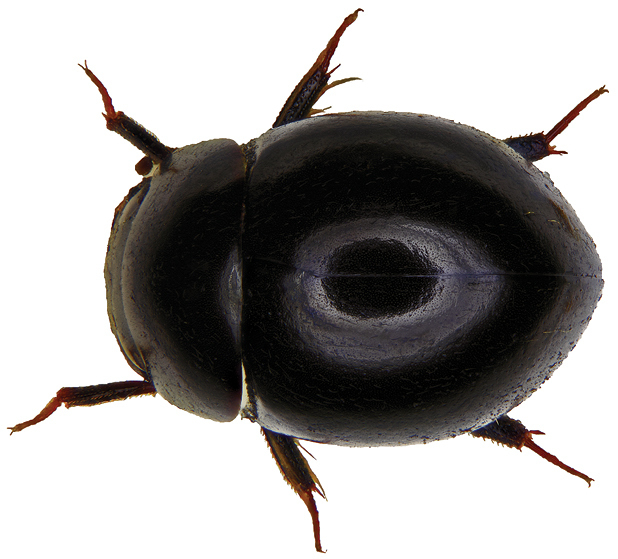
*Amphiops globus* Erichson, 1843 5.1 mm, iSimangaliso Wetland Park, False Bay (site 27), January 2015 DT Bilton, MS Bird & R Perissinotto leg.

**Figure 11.**
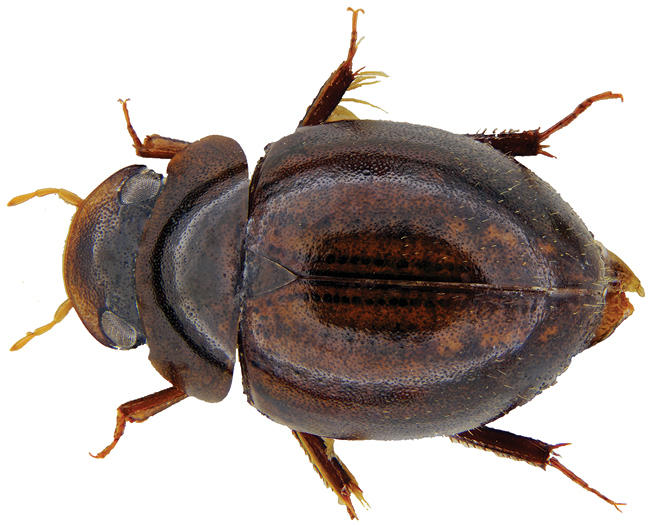
*Amphiops senegalensis* Laporte, 1840 5.0 mm, iSimangaliso Wetland Park, Eastern Shores (site 14), February 2015 DT Bilton, MS Bird & R Perissinotto leg.

**Figure 12.**
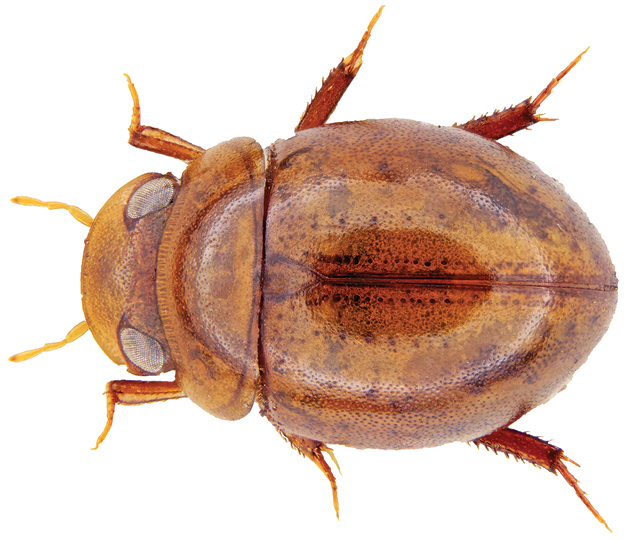
*Amphiops uhligi* Hebauer, 1995 3.5 mm, iSimangaliso Wetland Park, Eastern Shores (site 14), February 2015 DT Bilton, MS Bird & R Perissinotto leg.

**Figure 13.**
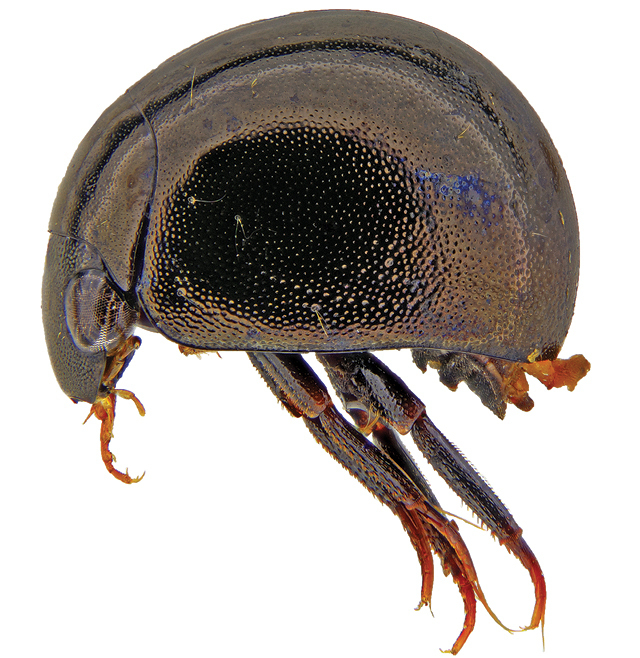
*Allocotocerus* sp. 4.3 mm, iSimangaliso Wetland Park, False Bay (site 27), February 2015 DT Bilton, MS Bird & R Perissinotto leg.

**Figure 14.**
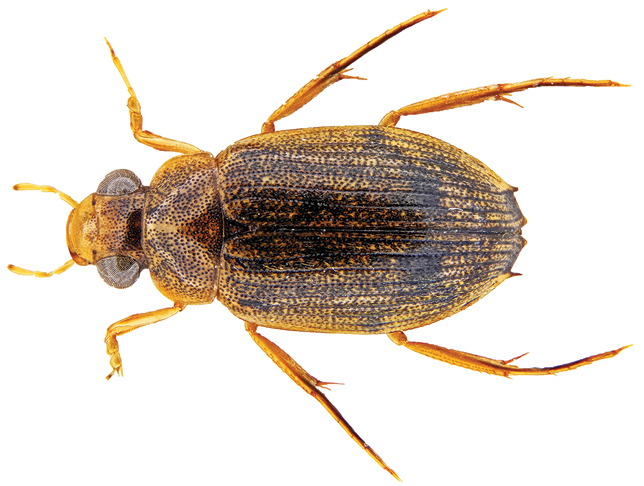
*Berosus cuspidatus* Erichson, 1843 5.2 mm, iSimangaliso Wetland Park, False Bay (site 29), February 2015 DT Bilton, MS Bird & R Perissinotto leg.

**Figure 15.**
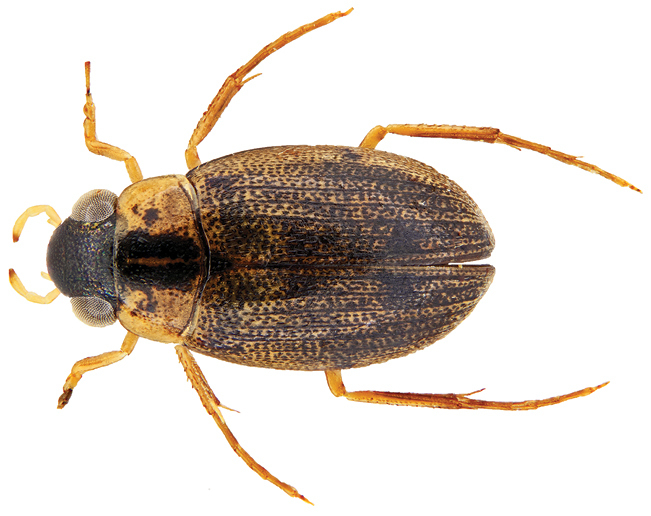
*Berosus viticollis* Boheman, 1851 2.8 mm, iSimangaliso Wetland Park, False Bay (site 29), February 2015 DT Bilton, MS Bird & R Perissinotto leg.

**Figure 16.**
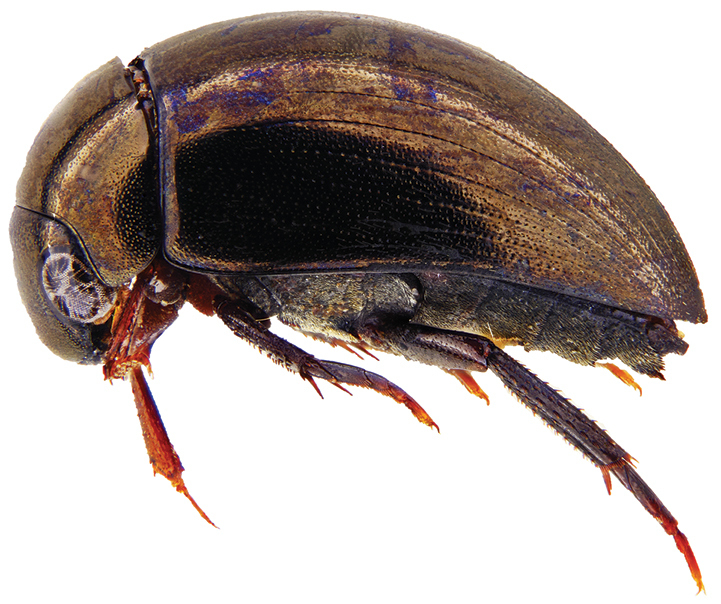
*Regimbartia nilotica* (Sharp, 1903) 5.4 mm, iSimangaliso Wetland Park, False Bay (site 27), January 2015 DT Bilton, MS Bird & R Perissinotto leg.

**Figure 17.**
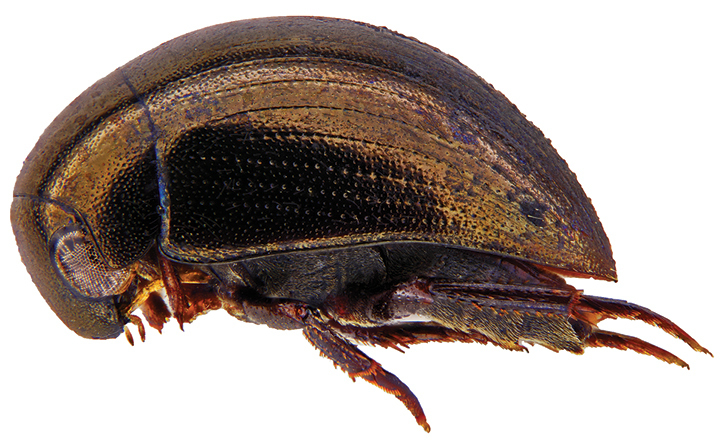
*Regimbartia obsoleta* (Régimbart, 1906) 3.8 mm, iSimangaliso Wetland Park, False Bay (site 27), January 2015 DT Bilton, MS Bird & R Perissinotto leg.

**Figure 18.**
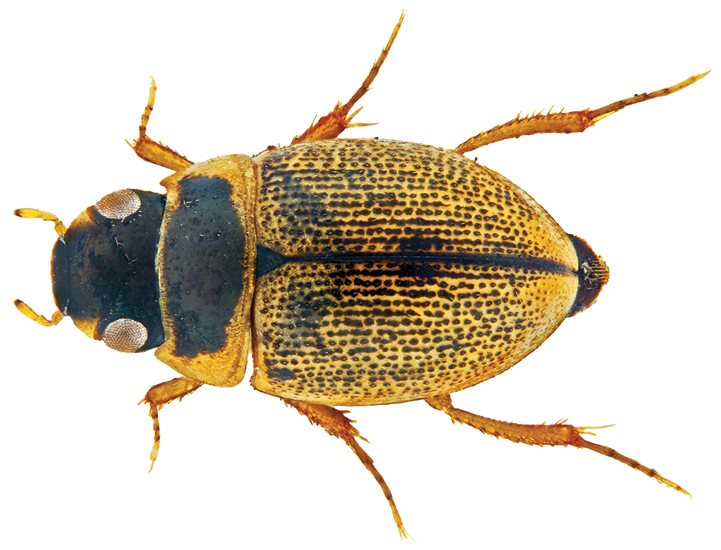
*Laccobius uhligi* Gentili, 1995 1.8 mm, iSimangaliso Wetland Park, Catalina Bay (site 32), February 2015 DT Bilton, MS Bird & R Perissinotto leg.

**Figure 19.**
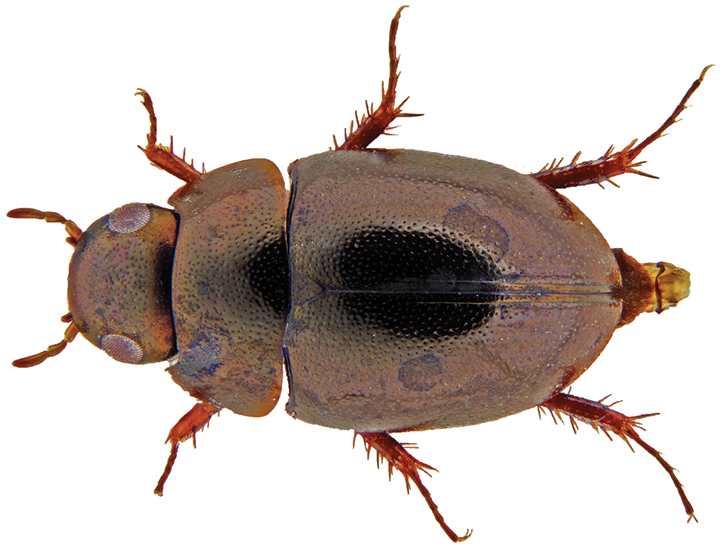
*Paracymus amplus* Wooldridge, 1977 2.5 mm, iSimangaliso Wetland Park, Catalina Bay (site 32), February 2015 DT Bilton, MS Bird & R Perissinotto leg.

**Figure 20.**
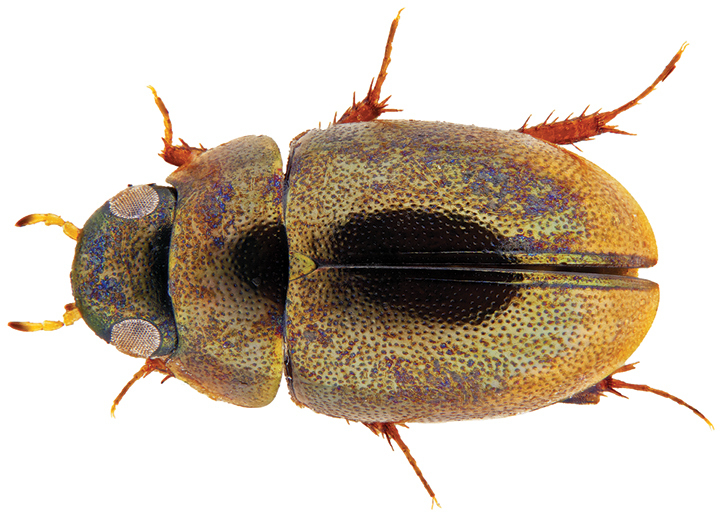
*Paracymus exiguus* Wooldridge, 1977 2.5 mm, iSimangaliso Wetland Park, False Bay (site 29), February 2015 DT Bilton, MS Bird & R Perissinotto leg.

**Figure 21.**
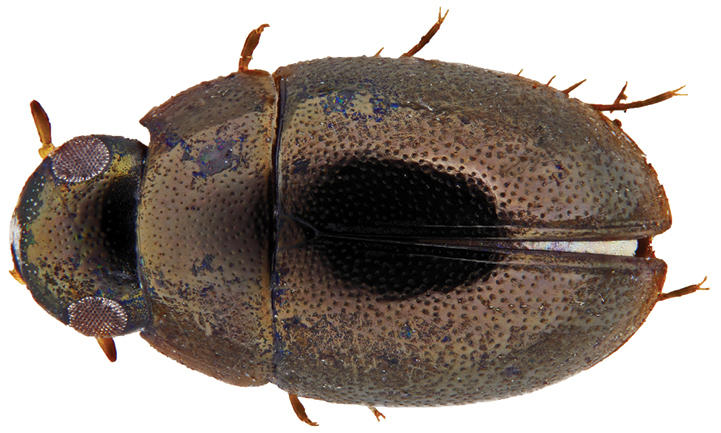
*Paracymus pisanus* Balfour-Browne, 1954 1.9 mm, iSimangaliso Wetland Park, False Bay (site 27), January 2015 DT Bilton, MS Bird & R Perissinotto leg.

**Figure 22.**
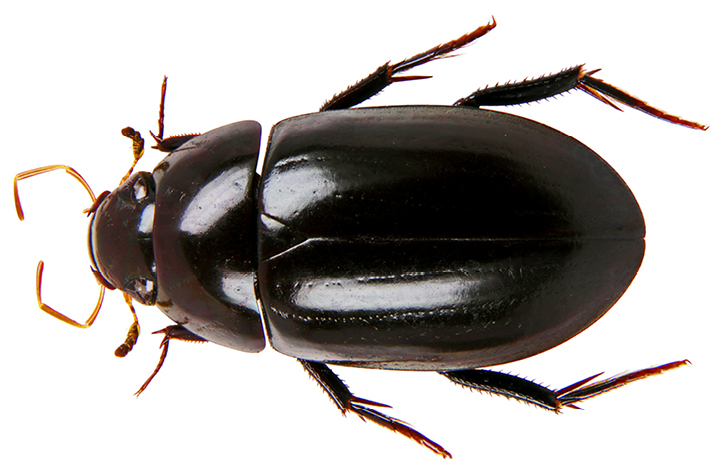
*Hydrobiomorpha perissinottoi* Bilton, 2016 18.5 mm, iSimangaliso Wetland Park, Eastern Shores (site 22), February 2015 DT Bilton, MS Bird & R Perissinotto leg.

**Figure 23.**
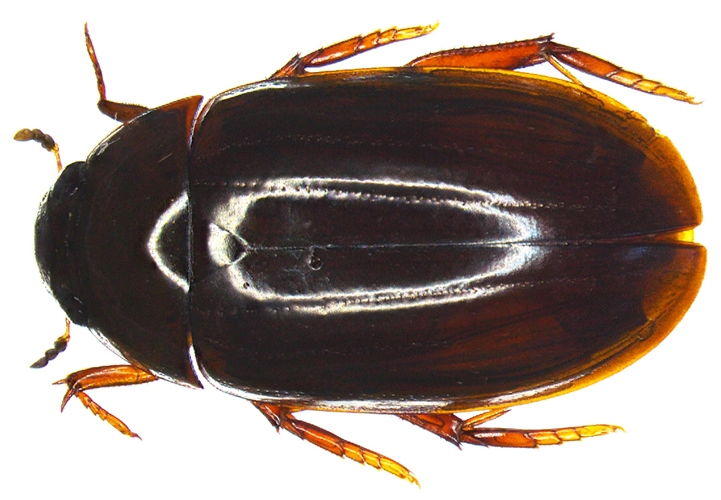
*Hydrochara elliptica* (Fabricius, 1801) 16.0 mm, iSimangaliso Wetland Park, False Bay (site 31), November 2013 MS Bird & R Perissinotto leg.

**Figure 24.**
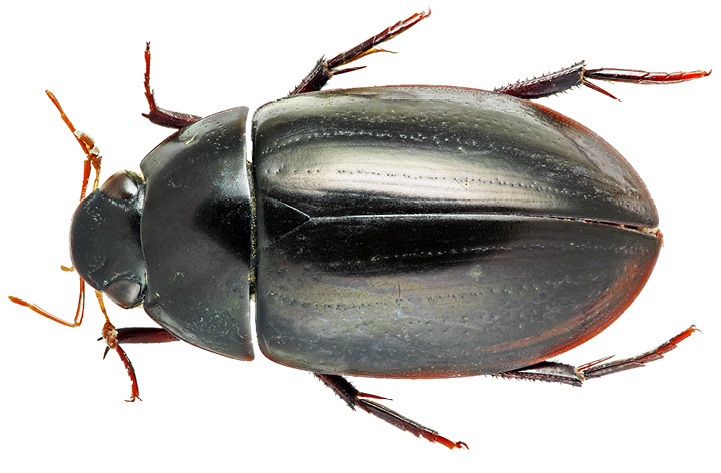
*Hydrochara fulvofemorata* (Fairmaire, 1869) 16.2 mm, iSimangaliso Wetland Park, False Bay (site 27), January 2015 DT Bilton, MS Bird & R Perissinotto leg.

**Figure 25.**
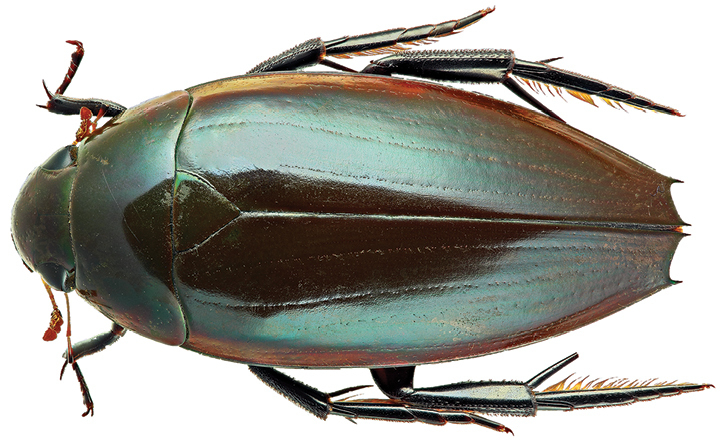
*Hydrophilus aculeatus* (Solier, 1834) 37.0 mm, iSimangaliso Wetland Park, Eastern Shores (site 14), February 2015 DT Bilton, MS Bird & R Perissinotto leg.

**Figure 26.**
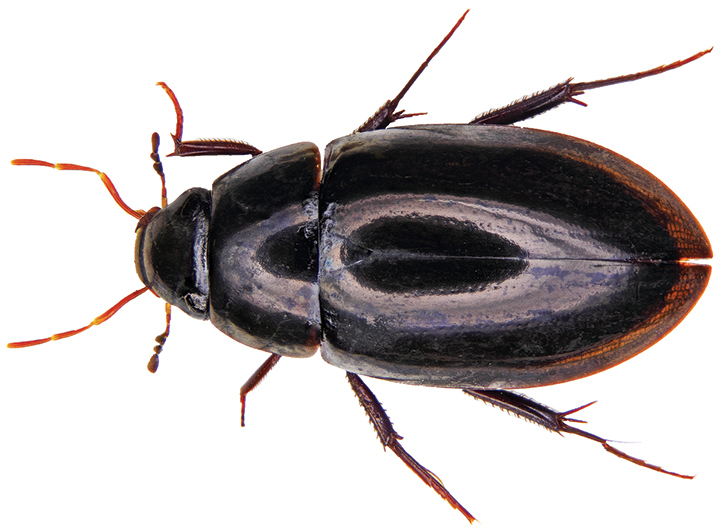
*Sternolophus solieri* Laporte, 1840 9.9 mm, iSimangaliso Wetland Park, False Bay (site 27), January 2015 DT Bilton, MS Bird & R Perissinotto leg.

**Figure 27.**
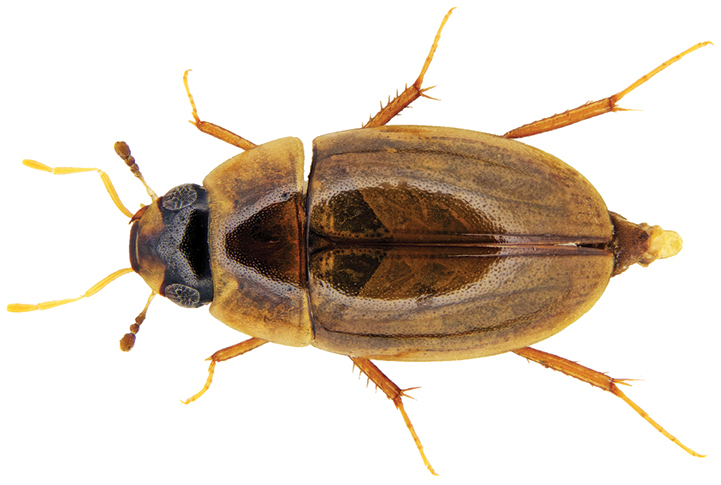
Enochrus (Methydrus) sp. 2.8 mm, iSimangaliso Wetland Park, Catalina Bay (site 32), February 2015 DT Bilton, MS Bird & R Perissinotto leg.

**Figure 28.**
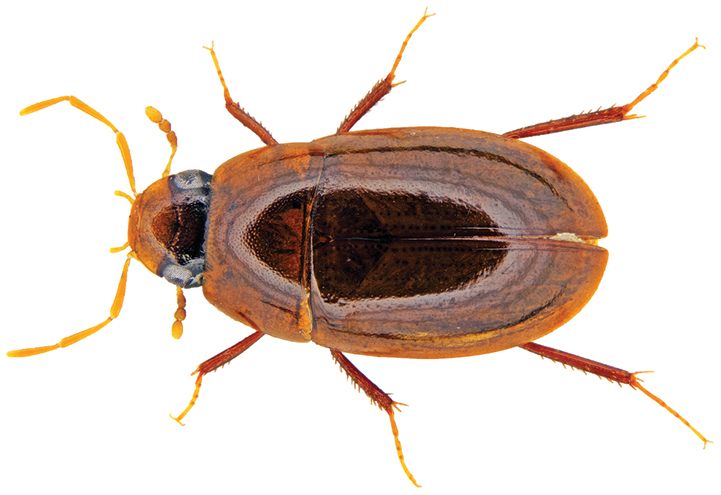
Chasmogenus cf. patrizii (Balfour-Browne, 1948) 3.4 mm, iSimangaliso Wetland Park, Eastern Shores (site 23), February 2015 DT Bilton, MS Bird & R Perissinotto leg.

**Figure 29.**
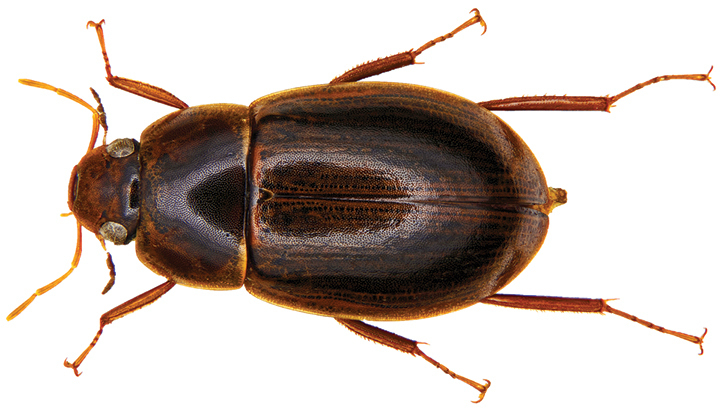
*Helochares dilutus* (Erichson, 1843) 6.8 mm, iSimangaliso Wetland Park, False Bay (site 27), January 2015 DT Bilton, MS Bird & R Perissinotto leg.

**Figure 30.**
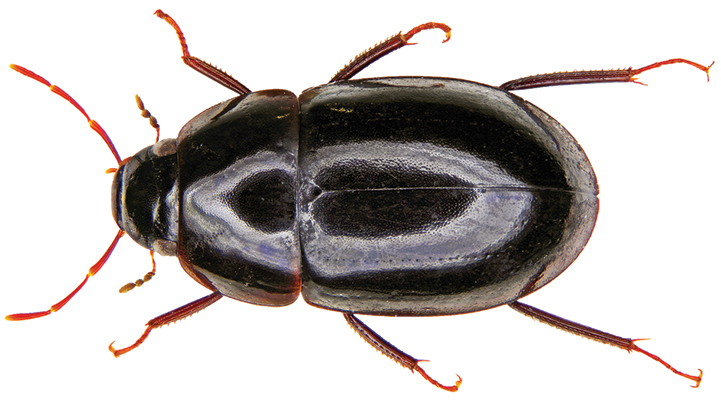
*Helochares longipalpis* (Murray, 1859) 7.0 mm, iSimangaliso Wetland Park, Catalina Bay (site 32), February 2015 DT Bilton, MS Bird & R Perissinotto leg.

**Figure 31.**
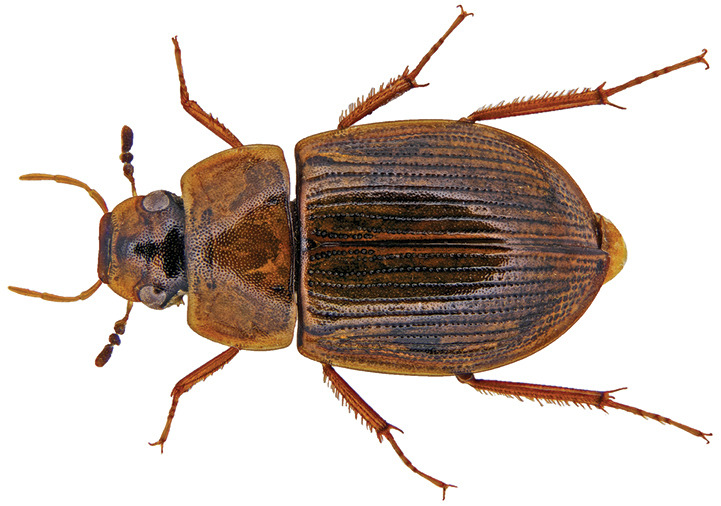
*Helochares* sp. 1 4.8 mm, iSimangaliso Wetland Park, False Bay (site 27), January 2015 DT Bilton, MS Bird & R Perissinotto leg.

**Figure 32.**
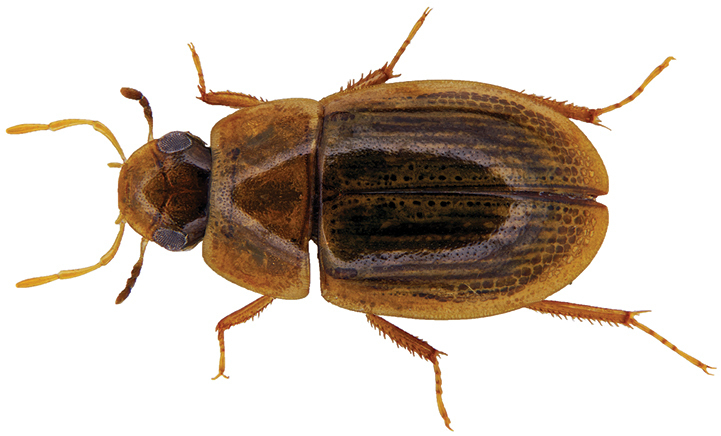
*Helochares* sp. 2 2.9 mm, iSimangaliso Wetland Park, False Bay (site 27), January 2015 DT Bilton, MS Bird & R Perissinotto leg.

**Figure 33.**
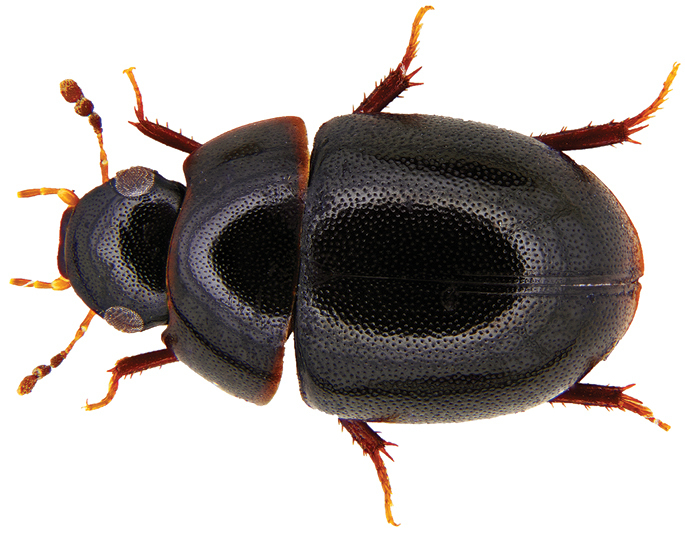
*Coelostoma* sp. 1 4.5 mm, iSimangaliso Wetland Park, Catalina Bay (site 32), February 2015 DT Bilton, MS Bird & R Perissinotto leg.

**Figure 34.**
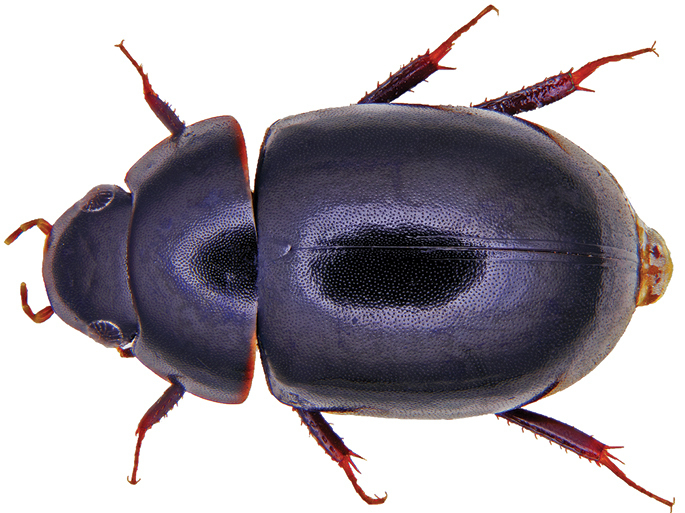
*Coelostoma* sp. 2 6.3 mm, iSimangaliso Wetland Park, Eastern Shores (site 23), February 2015 DT Bilton, MS Bird & R Perissinotto leg.

**Figure 35.**
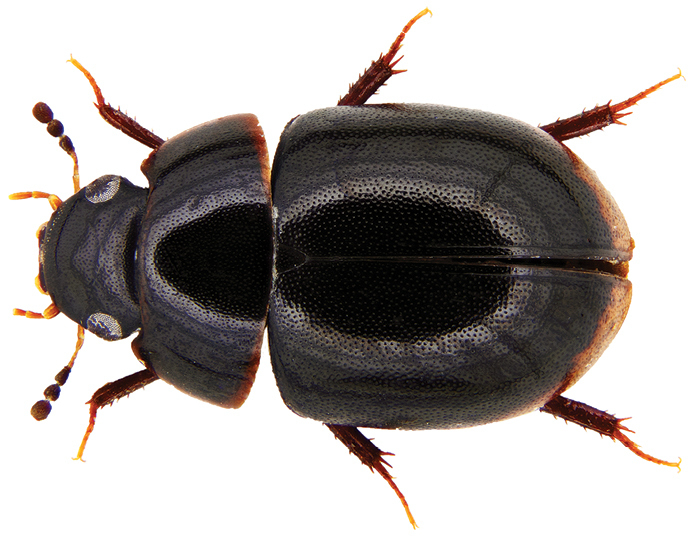
*Coelostoma* sp. 3 4.8 mm, iSimangaliso Wetland Park, Eastern Shores (site 23), February 2015 DT Bilton, MS Bird & R Perissinotto leg.

**Figure 36.**
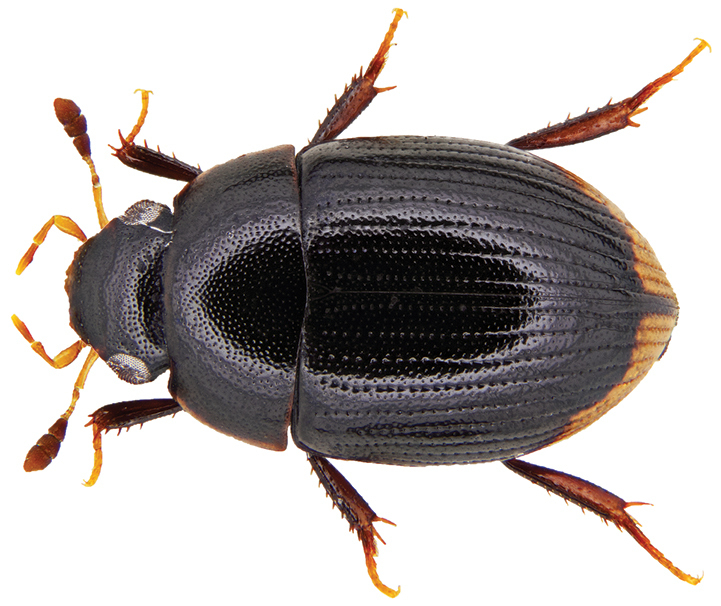
*Cercyon dieganus* Régimbart, 1903 2.6 mm, iSimangaliso Wetland Park, Eastern Shores (site 23), February 2015 DT Bilton, MS Bird & R Perissinotto leg.

**Figure 37.**
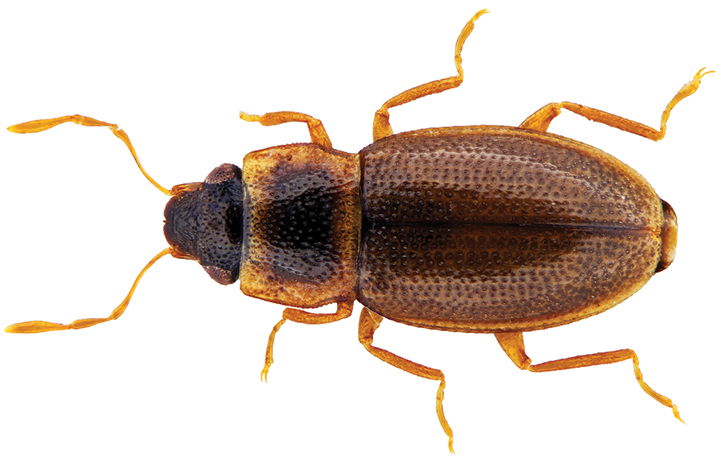
*Hydraena cooperi* Balfour-Browne, 1954 1.5 mm, iSimangaliso Wetland Park, Catalina Bay (site 32), February 2015 DT Bilton, MS Bird & R Perissinotto leg.

**Figure 38.**
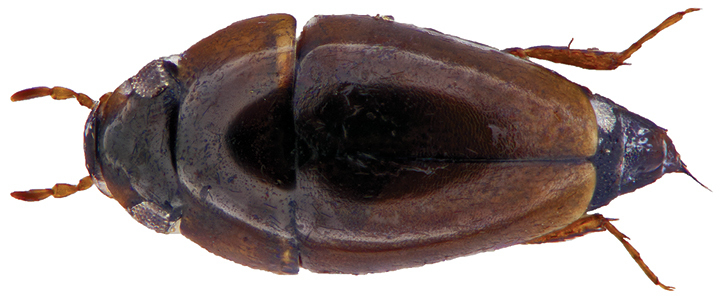
*Limnebius probus* Perkins, 2015 0.9 mm, iSimangaliso Wetland Park, False Bay (site 27), January 2015 DT Bilton, MS Bird & R Perissinotto leg.

**Figure 39.**
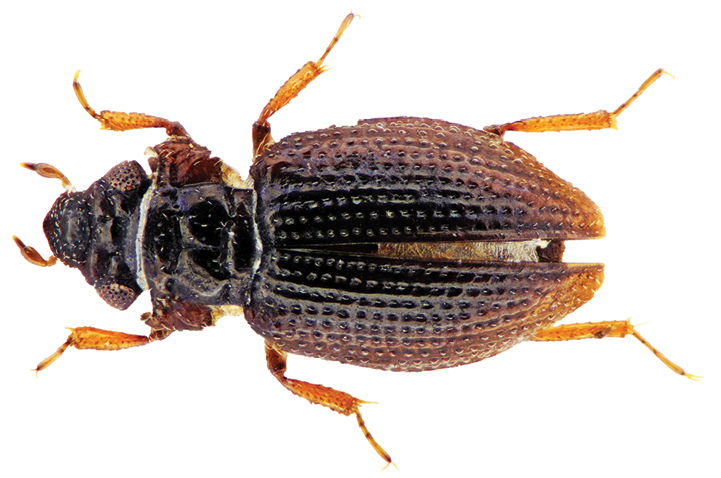
Aulachochthebius cf. continentalis (Orchymont, 1929) 1.2 mm, iSimangaliso Wetland Park, Eastern Shores (site 21), February 2015 DT Bilton, MS Bird & R Perissinotto leg.

**Figure 40.**
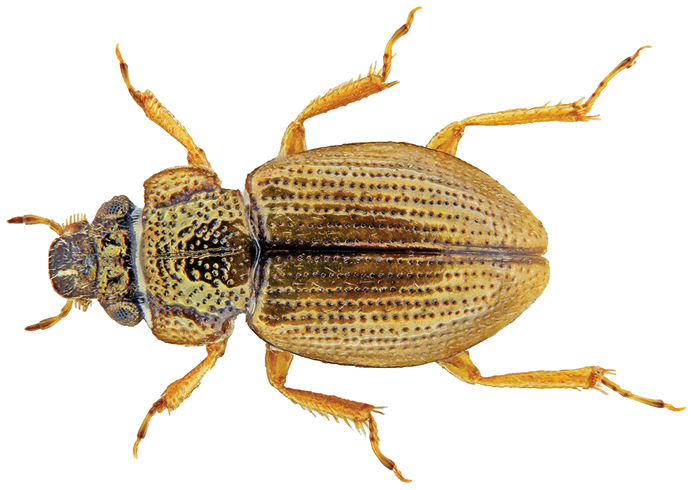
*Ochthebius andronicus* Orchymont, 1948 2.0 mm, iSimangaliso Wetland Park, Eastern Shores (site 21), February 2015 DT Bilton, MS Bird & R Perissinotto leg.

**Figure 41.**
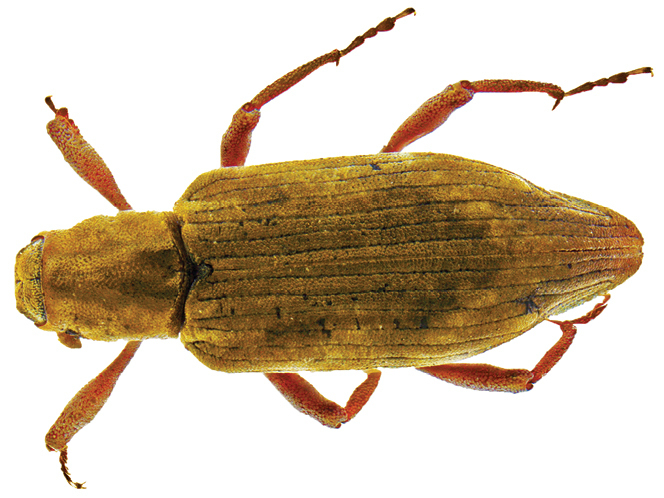
Pseudobagous cf. longulus (Gyllenhal, 1836) 5.0 mm, iSimangaliso Wetland Park, Eastern Shores (site 13), July 2014 MS Bird leg.

## Appendix I

Annotated and illustrated checklist of the Polyphaga of the Lake St Lucia system, 2013–2015.

The following list includes photographs of all species recorded during the dedicated water beetle surveys conducted by the authors during the period 2013 to 2015.

### Family: Hydrochidae

#### Hydrochus sp. 1

Taxon classification AnimaliaColeopteraHydrochidae

##### Remarks.

The Afrotropical species of this genus are in need of revision.

##### Distribution.

Range unknown. Afrotropical.

##### St Lucia records.

Recorded at Western Shores, Eastern Shores and False Bay in July 2014 and January/February 2015. Previously recorded in fresh water wetlands by Vrdoljak (2004) in 2002/2003.

#### Hydrochus sp. 2

Taxon classification AnimaliaColeopteraHydrochidae

##### Remarks.

The Afrotropical species of this genus are in need of revision.

##### Distribution.

Range unknown. Afrotropical.

##### St Lucia records.

Recorded at Western Shores, Eastern Shores and False Bay in January/February 2015. Previously recorded in fresh water wetlands by Vrdoljak (2004) in 2002/2003.

#### Hydrochus sp. 3

Taxon classification AnimaliaColeopteraHydrochidae

##### Remarks.

The Afrotropical species of this genus are in need of revision.

##### Distribution.

Range unknown. Afrotropical.

##### St Lucia records.

Recorded at Western Shores in January/February 2015. Previously recorded in fresh water wetlands on the Eastern Shores of Lake St Lucia by Vrdoljak (2004) in 2002/2003.

### Family: Spercheidae

#### Spercheus cerisyi

Taxon classification AnimaliaColeopteraSpercheidae

Guérin-Méneville, 1842

##### Synonyms.

*Spercheus crenaticollis* Régimbart, 1906, *Sphercheus* [!] *capicola* Péringuey, 1829, Spercheus cerisyi var. diminutus Hebauer, 1997

##### Remarks.

Ponds and other lentic waters, in vegetation.

##### Distribution.

Widespread to Western, Central and Eastern Africa and Madagascar; reaching the Palaearctic in Egypt, Iraq and Israel.

##### St Lucia records.

Recorded at Western Shores, Eastern Shores and False Bay in January/February 2015. Previously recorded at Dukandlovu by SANC – years not specified.

#### Spercheus senegalensis

Taxon classification AnimaliaColeopteraSpercheidae

Castelnau, 1832

##### Synonyms.

*Spercheus sulcatus* Gory, 1834, *Sphercheus* [!] *algoensis* Péringuey, 1892, *Spercheus distinguendus* Fairmaire, 1893.

##### Remarks.

Ponds and other lentic waterbodies rich in vegetation.

##### Distribution.

Widespread to Western, Central and Eastern Africa and Madagascar; apparently reaching the Palaearctic in Turkey.

##### St Lucia records.

Recorded at Western Shores, Eastern Shores and False Bay in January/February 2015. Previously recorded at St Lucia and Dukandlovu by SANC – years not specified.

### Family: Hydrophilidae

#### Amphiops globus

Taxon classification AnimaliaColeopteraHydrophilidae

Erichson, 1843

##### Remarks.

Ponds and other lentic waterbodies.

##### Distribution.

Widespread to Western, Central and Eastern Africa. Also reported from China.

##### St Lucia records.

Recorded at Western Shores, Eastern Shores and False Bay in January/February 2015.

#### Amphiops senegalensis

Taxon classification AnimaliaColeopteraHydrophilidae

(Laporte, 1840)

##### Synonyms.

*Amphiops lucidus* Erichson, 1843, *Cyprimorphus compressus* Fairmaire, 1873, *Amphiops Abeillei* Guillebeau, 1896, Amphiops lucidus var. abeillei Guillebeau, 1896, *Amphiops lasioides* Régimbart, 1903.

##### Remarks.

Ponds and other lentic waterbodies.

##### Distribution.

Widespread to Western, Central, Northern and Eastern Africa; reaching the Palaearctic in Egypt and Morocco.

##### St Lucia records.

Recorded at Western Shores, Eastern Shores and False Bay in July 2014 and January/February 2015.

#### Amphiops uhligi

Taxon classification AnimaliaColeopteraHydrophilidae

Hebauer, 1995

##### Remarks.

Found in dense vegetation in a small wetland at St Lucia.

##### Distribution.

Namibia, Botswana and Zambia. New record for South Africa.

##### St Lucia records.

Recorded at Eastern Shores in January/February 2015.

#### Allocotocerus sp.

Taxon classification AnimaliaColeopteraHydrophilidae

##### Remarks.

Species-level identification requires comparison with types.

##### Distribution.

Range unknown.

##### St Lucia records.

Recorded at Western Shores, Eastern Shores and False Bay in November 2013, July 2014 and January/February 2015. Previously recorded at fresh water wetlands on the Eastern Shores of Lake St Lucia by Vrdoljak (2004) in 2002/2003.

#### Berosus cuspidatus

Taxon classification AnimaliaColeopteraHydrophilidae

Erichson, 1843

##### Synonyms.

*Berosus bispinosus* Boheman, 1851, *Berosus acutispina* Fairmaire, 1869, Berosus cuspidatus ssp. acutispina Fairmaire, 1869, *Berosus gracilispina* Régimbart, 1906.

##### Remarks.

Ponds and lagoons, particularly with exposed substrate and some mineralisation/salinity.

##### Distribution.

Widespread to Western, Central and Eastern Africa and Madagascar and the Seychelles; reaching the Palaearctic in Egypt.

##### St Lucia records.

Recorded at Western Shores, Eastern Shores and False Bay in January/February 2015. Previously recorded at False Bay by Day et al. (1954) and Millard and Broekhuysen (1970) in 1948.

#### Berosus viticollis

Taxon classification AnimaliaColeopteraHydrophilidae

Boheman, 1851

##### Remarks.

Lentic waters, particularly with exposed substrates.

##### Distribution.

Widespread to Western, Central and Eastern Africa and Madagascar.

##### St Lucia records.

Recorded at Western Shores and False Bay in November 2013.

#### Regimbartia nilotica

Taxon classification AnimaliaColeopteraHydrophilidae

(Sharp, 1903)

##### Synonyms.

*Volvulus compressus* Régimbart, 1906, *Regimbartia compressa* (Régimbart, 1906).

##### Remarks.

Lentic waters, in vegetation.

##### Distribution.

Widespread to Western, Central and Eastern Africa; reaching the Palaearctic in Egypt.

##### St Lucia records.

Recorded at Western Shores, Eastern Shores and False Bay in November 2013 and January/February 2015.

#### Regimbartia obsoleta

Taxon classification AnimaliaColeopteraHydrophilidae

(Régimbart, 1906)

##### Remarks.

Lentic waters, in vegetation.

##### Distribution.

Widespread to Western, Central and Eastern Africa.

##### St Lucia records.

Recorded at Western Shores, Eastern Shores and False Bay in January/February 2015.

#### Laccobius uhligi

Taxon classification AnimaliaColeopteraHydrophilidae

Gentili, 1995

##### Remarks.

Seepages over peat beside lagoon at St Lucia.

##### Distribution.

Namibia (Caprivi Strip) and Botswana (Okavango). New record for South Africa.

##### St Lucia records.

Recorded at Eastern Shores in January/February 2015.

#### Paracymus amplus

Taxon classification AnimaliaColeopteraHydrophilidae

Wooldridge, 1977

##### Remarks.

Lentic waters, in vegetation.

##### Distribution.

A species currently only known from South Africa.

##### St Lucia records.

Recorded at Eastern Shores in January/February 2015.

#### Paracymus exiguus

Taxon classification AnimaliaColeopteraHydrophilidae

Wooldridge, 1977

##### Remarks.

Lentic waters, in vegetation.

##### Distribution.

Described from Zimbabwe. New record for South Africa.

##### St Lucia records.

Recorded at Western Shores, Eastern Shores and False Bay in January/February 2015.

#### Paracymus pisanus

Taxon classification AnimaliaColeopteraHydrophilidae

Balfour-Browne, 1954

##### Remarks.

Lentic waters, in vegetation.

##### Distribution.

South Africa, Namibia and Botswana.

##### St Lucia records.

Recorded at Western Shores, Eastern Shores and False Bay in January/February 2015.

#### Hydrobiomorpha perissinottoi

Taxon classification AnimaliaColeopteraHydrophilidae

Bilton, 2016

##### Remarks.

A new species, first detected during this survey. Most close morphologically to *Hydrobiomorpha occidentalis* Balfour-Browne, 1939 from Nigeria and Sudan.

##### Distribution.

Currently only recorded from St Lucia – wider distribution unknown.

##### St Lucia records.

Recorded at Western Shores, Eastern Shores and False Bay in January/February 2015.

#### Hydrochara elliptica

Taxon classification AnimaliaColeopteraHydrophilidae

(Fabricius, 1801)

##### Synonyms.

*Hydrochares ellipticus* (Fabricius, 1801), *Hydrous uniformis* Fairmaire, 1869, Hydrophilus fulvo-femorata var. uniformis (Fairmaire, 1869).

##### Remarks.

Lentic waters, in vegetation.

##### Distribution.

Widespread to western, central and eastern Africa and Madagascar.

##### St Lucia records.

Recorded at False Bay in November 2013. Previously recorded at Dukandlovu Pan by the authors and deposited at UKZN in 2012.

#### Hydrochara fulvofemorata

Taxon classification AnimaliaColeopteraHydrophilidae

(Fairmaire, 1869)

##### Remarks.

Lentic waters, in vegetation.

##### Distribution.

Mozambique to Eastern Africa and Madagascar. New record for South Africa, first reported by Bilton (2016).

##### St Lucia records.

Recorded at Western Shores and False Bay in November 2013 and January/February 2015.

#### Hydrophilus aculeatus

Taxon classification AnimaliaColeopteraHydrophilidae

(Solier, 1834)

##### Synonyms.

*Hydrophilus spinipennis* Gory, 1834, *Hydrophilus armatus* Castelnau, 1840, *Hydrophilus lugubris* Motschulsky, 1845, *Hydrophilus aegyptiacus* Peyron, 1856.

##### Remarks.

Lentic waters, in vegetation.

##### Distribution.

Widespread to Western, Central and Eastern Africa, the Mascarenes and Arabia; reaching the Palaearctic in Egypt, Iran, Israel, Syria and Turkey.

##### St Lucia records.

Recorded at Western Shores, Eastern Shores and False Bay November 2013, July 2014 and January/February 2015. Previously recorded at False Bay by the authors and deposited at UNKZ in 2012.

#### Sternolophus solieri

Taxon classification AnimaliaColeopteraHydrophilidae

Laporte, 1840

##### Synonyms.

*Sternolophus rufipes* Solier, 1834, *Helobius noticollis* Mulsant, 1851, *Hydrous aeratus* Reiche & Saulcy, 1854, *Hydrous graecus* Baudi, 1864, *Sternolophus punctatulus* Schaufuss, 1883.

##### Remarks.

Lentic waters, in vegetation.

##### Distribution.

Widespread to Western, Central and Eastern Africa, Madagascar, the Cape Verdes, the Comoros; reaching the Palaearctic in Algeria, Egypt, Greece and Israel.

##### St Lucia records.

Recorded at Western Shores, Eastern Shores and False Bay in November 2013 and January/February 2015. Previously recorded at False Bay by the authors and deposited at UKZN in 2008.

#### Enochrus (Methydrus) sp.

Taxon classification AnimaliaColeopteraHydrophilidae

##### Remarks.

Lentic waters, in vegetation. African fauna requires revision.

##### Distribution.

Unknown.

##### St Lucia records.

Recorded at Western Shores, Eastern Shores and False Bay in November 2013, July 2014 and January/February 2015.

#### Chasmogenus cf. patrizii

Taxon classification AnimaliaColeopteraHydrophilidae

(Balfour-Browne, 1948)

##### Remarks.

Lentic waters, in vegetation. African fauna requires revision before certain identification can be reached.

##### Distribution.

Widespread to Central and Eastern Africa.

##### St Lucia records.

Recorded at Eastern Shores and False Bay in January/February 2015.

#### Helochares dilutus

Taxon classification AnimaliaColeopteraHydrophilidae

(Erichson, 1843)

##### Synonyms.

*Helochares niloticus* Sharp, 1903.

##### Remarks.

Lentic waters, in vegetation. Records from South Africa, Namibia, Madagascar and the Mascarenes have been referred to ssp. consputus Boheman, 1851.

##### Distribution.

Widespread to Western, Central and Eastern Africa and Madagascar.

##### St Lucia records.

Recorded at Western Shores, Eastern Shores and False Bay in July 2014 and January/February 2015. Previously recorded at St Lucia and Dukandlovu by SANC – years not specified.

#### Helochares longipalpis

Taxon classification AnimaliaColeopteraHydrophilidae

(Murray, 1859)

##### Remarks.

Lentic waters, in vegetation.

##### Distribution.

Widespread to Western, central and Eastern Africa; reaching the Palaearctic in Egypt and Israel.

##### St Lucia records.

Recorded at Western Shores, Eastern Shores and False Bay in July 2014 and January/February 2015. Previously recorded at St Lucia and Dukandlovu by SANC – years not specified.

#### Helochares sp. 1

Taxon classification AnimaliaColeopteraHydrophilidae

##### Remarks.

Lentic waters, in vegetation. The African *Helochares* fauna requires a thorough revision.

##### Distribution.

Unknown.

##### St Lucia records.

Recorded at Eastern Shores and False Bay in July 2014 and January/February 2015. Previously recorded at fresh water wetlands on the Eastern Shores of Lake St Lucia by Vrdoljak (2004) in 2002/2003.

#### Helochares sp. 2

Taxon classification AnimaliaColeopteraHydrophilidae

##### Remarks.

Lentic waters, in vegetation. The African *Helochares* fauna requires a thorough revision.

##### Distribution.

Unknown.

##### St Lucia records.

Recorded at Western Shores, Eastern Shores and False Bay in November 2013, July 2014 and January/February 2015. Previously recorded at fresh water wetlands on the Eastern Shores of Lake St Lucia by Vrdoljak (2004) in 2002/2003.

#### Coelostoma sp. 1

Taxon classification AnimaliaColeopteraHydrophilidae

##### Remarks.

The African fauna of this genus requires revision.

##### Distribution.

Unknown.

##### St Lucia records.

Recorded at Eastern Shores and False Bay in January/February 2015. Previously recorded at fresh water wetlands on the Eastern Shores of Lake St Lucia by Vrdoljak (2004) in 2002/2003.

#### Coelostoma sp. 2

Taxon classification AnimaliaColeopteraHydrophilidae

##### Remarks.

The African fauna of this genus requires revision.

##### Distribution.

Unknown.

##### St Lucia records.

Recorded at Western Shores, Eastern Shores and False Bay in November 2013, July 2014 and January/February 2015. Previously recorded at fresh water wetlands on the Eastern Shores of Lake St Lucia by Vrdoljak (2004) in 2002/2003.

#### Coelostoma sp. 3

Taxon classification AnimaliaColeopteraHydrophilidae

##### Remarks.

The African fauna of this genus requires revision.

##### Distribution.

Unknown.

##### St Lucia records.

Recorded at Eastern Shores in January/February 2015. Previously recorded at fresh water wetlands on the Eastern Shores of Lake St Lucia by Vrdoljak (2004) in 2002/2003.

#### Cercyon dieganus

Taxon classification AnimaliaColeopteraHydrophilidae

Régimbart, 1903

##### Remarks.

In wet decaying vegetable debris in the margins of lentic waterbodies.

##### Distribution.

Widespread in Afrotropical Region, including Madagascar.

##### St Lucia records.

Recorded at Western Shores, Eastern Shores and False Bay in January/February 2015.

### Family: Hydraenidae

#### Hydraena cooperi

Taxon classification AnimaliaColeopteraHydraenidae

Balfour-Browne, 1954

##### Remarks.

Shallow margins of lentic and lotic waters.

##### Distribution.

Widespread in South Africa and also recorded from Angola and Namibia.

##### St Lucia records.

Recorded at Western Shores, Eastern Shores and False Bay in January/February 2015.

#### Limnebius probus

Taxon classification AnimaliaColeopteraHydraenidae

Perkins, 2015

##### Remarks.

Pond margins.

##### Distribution.

Eastern South Africa. Species recently described, but so far only known from South Africa.

##### St Lucia records.

Recorded at False Bay in January/February 2015.

#### Aulachochthebius cf. continentalis

Taxon classification AnimaliaColeopteraHydraenidae

(Orchymont, 1929)

##### Remarks.

Pond margins. Afrotropical species of the genus currently in revision (Perkins, pers. comm.).

##### Distribution.

Kenya. Most likely new to South Africa, regardless of species.

##### St Lucia records.

Recorded at Eastern Shores and False Bay in January/February 2015.

#### Ochthebius andronicus

Taxon classification AnimaliaColeopteraHydraenidae

Orchymont, 1948

##### Remarks.

Pond margins.

##### Distribution.

Widespread in Southern and Eastern Africa.

##### St Lucia records.

Recorded at Eastern Shores and False Bay in January/February 2015.

### Family: Curculionidae

#### Pseudobagous cf. longulus

Taxon classification AnimaliaColeopteraCurculionidae

(Gyllenhal, 1836)

##### Remarks.

In vegetation in lentic waterbodies.

##### Distribution.

Widespread in Southern Africa.

##### St Lucia records.

Recorded at Eastern Shores and False Bay in July 2014 and January/February 2015.
